# *In silico* identification and functional prediction of differentially expressed genes in South Asian populations associated with type 2 diabetes

**DOI:** 10.1371/journal.pone.0294399

**Published:** 2023-12-14

**Authors:** Md. Golam Rabby, Md. Hafizur Rahman, Md. Numan Islam, Md. Mostafa Kamal, Mrityunjoy Biswas, Mantasa Bonny, Md. Mahmudul Hasan

**Affiliations:** 1 Department of Nutrition and Food Technology, Jashore University of Science and Technology, Khulna, Bangladesh; 2 Department of Agro Product Processing Technology, Jashore University of Science and Technology, Khulna, Bangladesh; 3 Faculty of Food Sciences and Safety, Department of Quality Control and Safety Management, Khulna Agricultural University, Khulna, Bangladesh; The First Hospital of Jilin University, CHINA

## Abstract

Type 2 diabetes (T2D) is one of the major metabolic disorders in humans caused by hyperglycemia and insulin resistance syndrome. Although significant genetic effects on T2D pathogenesis are experimentally proved, the molecular mechanism of T2D in South Asian Populations (SAPs) is still limited. Hence, the current research analyzed two Gene Expression Omnibus (GEO) and 17 Genome-Wide Association Studies (GWAS) datasets associated with T2D in SAP to identify DEGs (differentially expressed genes). The identified DEGs were further analyzed to explore the molecular mechanism of T2D pathogenesis following a series of bioinformatics approaches. Following PPI (Protein-Protein Interaction), 867 potential DEGs and nine hub genes were identified that might play significant roles in T2D pathogenesis. Interestingly, CTNNB1 and RUNX2 hub genes were found to be unique for T2D pathogenesis in SAPs. Then, the GO (Gene Ontology) showed the potential biological, molecular, and cellular functions of the DEGs. The target genes also interacted with different pathways of T2D pathogenesis. In fact, 118 genes (including HNF1A and TCF7L2 hub genes) were directly associated with T2D pathogenesis. Indeed, eight key miRNAs among 2582 significantly interacted with the target genes. Even 64 genes were downregulated by 367 FDA-approved drugs. Interestingly, 11 genes showed a wide range (9–43) of drug specificity. Hence, the identified DEGs may guide to elucidate the molecular mechanism of T2D pathogenesis in SAPs. Therefore, integrating the research findings of the potential roles of DEGs and candidate drug-mediated downregulation of marker genes, future drugs or treatments could be developed to treat T2D in SAPs.

## Introduction

Type 2 diabetes (T2D) is a complex metabolic disorder that has sparked much research interest globally. In 2021, 536.6 million people had diabetes, and 783.2 million are expected to have T2D by 2045 [1]. Indeed, diabetes cases are also expected to rise among Southeast Asian populations over the next two decades, from 90.2 million in 2021 to 151.5 million people by 2045 [1]. In 2019, 1.5 million people worldwide died only due to diabetes [2]. In South Asia, Bangladeshi, Pakistani, Nepali, Bhutanese, Maldivian, Sri Lankan, and Singaporean populations showed a significant increase in T2D patients during the last three decades [3]. However, Indian populations showed the highest prevalence of diabetes, followed by the Chinese population. In 2025, a case of 69.9 million diabetes is anticipated in India, where the majority are still undiagnosed clinically [4]. Certainly, more than 6.3 million Pakistani populations are suffering from diabetes [5]. In the Singaporean population, one-third are at risk of diabetes during the whole life span, and the number is anticipated to be increased by more than 1 million by 2050 [6]. Similarly, during the last three decades, the prevalence of T2D has significantly increased in Nepali, Sri Lankan, Bhutanese, and Maldivian populations. Not surprisingly, T2D is increasing alarmingly among other SAPs, especially in Bangladesh. Based on age, sex, and disease complexities, the overall prevalence of T2D in the Bangladeshi population ranges from 2.21 to 35% [7], which is very closed to India and China. In 2021, 13.1 million Bangladeshi adults had diabetes, and the cases are anticipated to be 22.3 million by 2045 [1].

T2D is the cumulative effect of genetic and environmental factors. Multiple susceptible genetic signatures responsible for T2D have already been identified in various South Asian countries using genome-wide association studies (GWAS) [8]. In South Asian population (SAP), diverse genetic variation, population structure, and disease associations led to inconsistent population-specific medical treatment. Hence, the construction of reference genome databases for specific populations and GWAS among various populations are urgently needed [9].

Due to considerable intergroup cultural differences, SAP possesses a significant genetic diversity [10]. Research on Asian human pathogenomics will guide physicians to suggest precise medication for the respective population [11,12]. Among SAPs, significant genetic diversity was observed among different ethnicities in the Singaporean population (Chinese, Malay, and Indian). Additionally, 14 potential loci were identified in various Asian and Oceanian populations with solid relationships with complex traits and disorders [11]. Consequently, research on human pathogenomics consequences the availability of data on human genetics over a wide range of geographical distribution. [11,13].

Indeed, several genes have already been identified that are associated with T2D pathogenesis in SAP [4,5,14]. In Indian and Pakistani populations, *TCF7L2*, *FTO*, *PPARG2*, *IRS1*, *SLC30A8*, *CDKN2A*, *HHEX*, *CDKAL1*, *EXT2*, *ADIPOQ*, *IGF2BP2*, *WFS1*, *LOC387761*, *CAPN10*, *CDKN2B*, *MTHFR*, *KCNJ11*, *SGCG*, *ADAM30*, *THADA*, *GCK*, *LOC646279*, *TCF-2/ HNF1B*, *NOTCH2*, *VEGFA*, *and HOMA-β* genes were found to be associated with T2D pathogenesis [3]. Among SAP, HNF4A, HMG20A, VPS26A, GRB14, AP3S2, and ST6GAL1loci were found to be associated with T2D. Interestingly, Single Nucleotide Polymorphisms (SNPs) at GRB14, HNF4A, and ST6GAL1 genes were also associated with pancreatic beta-cell function and insulin sensitivity, respectively [15].

Although different genes and loci have already been identified for T2D pathogenesis among SAP, how these genes interact at transcript and protein levels in disease association, different metabolic pathways, biological systems, and drug interaction are still unknown that needs to be elucidated. In addition, extensive genetic diversity among different SAP guides further research on population-specific disease associations [8]. Bioinformatic research facilitates analyzing variations in gene expression at the transcript level, which guides to identify of differentially expressed genes (DEGs) and their functional role in T2D pathogenesis [16]. Thus, the identification and functional prediction of DEGs, following their role in different metabolic pathways associated with type T2D pathogenesis would be a significant advancement of T2D research in SAP.

Therefore, we have designed the research to identify and predict the functions of DEGs associated with T2D pathogenesis in SAP using GWAS catalog data and gene expression omnibus (GEO) data. These screened DEGs were utilized for further analyses following protein-protein interaction, gene ontology, pathway enrichment, miRNA target regulatory, disease association, and drug-gene interaction to elucidate the mechanisms of T2D pathogenesis among SAPs.

## Methods

### Microarray data

The GEO is a publicly available functional genomic resource that comprises information from chips, microarrays, and high-throughput gene expression investigations. Two microarray datasets (GSE26168 and GSE78721) were obtained from the NCBI database called the GEO database (https://www.ncbi.nlm.nih.gov/gds), and each GSE file was separated into control and disease states. Both datasets were from the South Asian populations (Singaporean and Indian). The dataset of the Singaporean population (GSE26168) is based on the GPL6883 platform, and we have used eight controls and nine T2D-affected samples within 60 samples of the dataset. While the dataset of the Indian population (GSE78721) is based on the GPL15207 platform, and we have used 16 controls and 19 T2D-affected samples within 130 samples of the dataset. The differentially expressed genes (DEGs) in T2D-affected populations were identified using the GEO2R (http://www.ncbi.nlm.nih.gov/geo/geo2r) database. GEO2R is a web-based application that analyzes two or more GEO datasets to elucidate the DEGs under various experimental conditions. The modified Benjamin and Hochberg’s false discovery rate and the P-values were utilized to balance the identification of statistically significant DEGs with limitations of false positives. DEGs in T2D were identified using the fold change value, |log FC| > 1.5 and adj. P 0.05 [17–21].

### Genome-wide association study (GWAS)

The publicly available GWAS catalog database (https://www.ebi.ac.uk/gwas/) was used to explore the DEGs associated with T2D in SAP. The database is used to analyze SNP-trait correlations for observing DEGs and SNPs associated with different diseases. We have chosen 17 GWAS catalog datasets of T2D (GCST002352, GCST001213, GCST008833, GCST004894, GCST001033, GCST001759, GCST001809, GCST005414, GCST010557, GCST010553, GCST007515, GCST007516, GCST006867, GCST010272, GCST011337, GCST011329, GCST011321) among SAPs where, overlapping genes were omitted [22].

### Protein-protein interaction (PPI) network analysis and identification of Hub genes

The PPI of the translated proteins of identified DEGs was constructed using the publicly available STRING database (https://string-db.org/). After inputting the ID of the identified DEGs onto the STRING database, the species "*Homo sapiens*" was selected, and the high confidence (0.900) interaction score was set to create the PPI interaction network. Subsequently, Cytoscape software was used to visualize the PPI networks [23–25]. Then, we have used MCODE (Molecular Complex Detection) (http://apps.cytoscape.org/apps/mcode) and Cytohubba on Cytoscape (http://apps.cytoscape.org/apps/cytohubba) plugins to determine the most critical subnetwork modules. Cytohubba follows topological algorithms to visualize protein associations. MCODE gives clusters of sub-networks. We have set Node Score Cutoff = 0.2, Degree Cutoff = 2, and K-Score = 2 during analysis in MCODE [26].

### Gene ontology (GO) enrichment analysis

The GO analysis was done using the ToppFun tool of ToppGene (https://toppgene.cchmc.org/enrichment.jsp) to conduct the functional enrichment of DEGs. We have used the default settings of the ToppGene suite portal with the p-value of 0.05 with corrected an FDR value. In the default setting, Correction value = FDR, p-Value cutoff score = 0.05, Gene Limits 1< = n< = 2000 were maintained. Then, the top ten significant roles in cellular components, biological processes, and molecular functions were presented [27].

### Pathway enrichment analysis

The publicly available Web-Gestalt (WEB-based Gene SeT AnaLysis Toolkit) (http://www.webgestalt.org/) database was used to analyze the KEGG pathways enrichment, setting the FDR (false discovery rate) value of 0.05 as the cutoff value [28]. The pathway enrichment analysis was done following the Over Representation Analysis (ORA) method, which is one of the three WebGestalt software methods. *Homo sapiens* was the reference genome during the KEGG pathway enrichment analysis, where Gene Symbol ID was used as the gene ID [29].

### Construction of DEGs-miRNA regulatory network

The publicly available miRNet (https://www.mirnet.ca/) database was used to predict the regulatory network of the identified DEGs-miRNAs associated with T2D. In the database, the genes option was selected and gene IDs were inputted to identify DEGs-miRNA association for T2D following the default setting of *Homo sapiens* species. The regulatory network of DEGs-miRNA was developed and visualized using Cytoscape. The top 30 degrees of the node was chosen to visualize the DEGs-miRNAs regulatory network [30].

### Disease association analysis

The Web-Gestalt database (http://www.webgestalt.org/) was used to analyze disease associations of the DEGs following the default setting of the *Homo sapiens* genome. Web-Gestalt database uses Benjamini and Hochberg approach and a hypergeometric statistical test to determining the false discovery rate. The top ten most significant disease associations were presented [31].

### Construction of candidate drug-gene interaction network

The interaction networks of candidate drug-gene were predicted using the publicly available DGIdb database (Drug Gene Interaction Database) (DGIdb, v3.0.2, https://www.dgidb.org/). The candidate drug-gene interaction pairs were obtained using known and FDA-approved drugs. Following that, Cytoscape software was used to analyze and visualize the drug-gene interaction network [32].

## Results

### Identification of differentially expressed genes (DEGs) from the microarray GEO dataset

The GEO database was used to identify DEGs associated with T2D. Two South Asian GEO datasets (GSE26168 and GSE78721) were analyzed. Here, we have found 221 DEGs in the GSE26168 (GPL6883) dataset (The CDC42 gene showed both up and down-regulated), including 95 upregulated genes and 127 downregulated genes. In the GSE78721 (GPL15207) dataset, 28 DEGs were found, where 26 genes were upregulated and two were downregulated. In the Venn diagram, no overlapped gene was observed in these two datasets. Finally, 249 DEGs were detected in the selected GEO datasets (S1 Table).

### Genome-wide association study (GWAS)

In 17 GWAS catalog datasets, 1378 DEGs were found to be associated with T2D in SAP. All the DEGs found in the 17 GWAS catalog datasets are as follows: 76 DEGs in GCST002352 datasets, 7 DEGs in GCST001213 datasets, 24 DEGs in GCST008833 datasets, 111 DEGs in GCST004894 datasets, 18 DEGs in GCST001033 datasets, 7 DEGs in GCST001759 datasets, 12 DEGs in GCST001809 datasets, 33 DEGs in GCST005414 datasets, 695 DEGs in GCST010557 datasets, 107 DEGs in GCST010553 datasets, 36 DEGs in GCST007515 datasets, 34 DEGs in GCST007516 datasets, 174 DEGs in GCST006867 datasets, 15 DEGs in GCST010272 datasets, 12 DEGs in GCST011337 datasets, 14 DEGs in GCST011329 datasets and 3 DEGs in GCST011321 datasets. After omitting overlapped genes, 843 unique genes were found to be associated with T2D pathogenesis in SAP (S2 Table).

### Construction of PPI network and Hub genes identification

**Fig 1:**
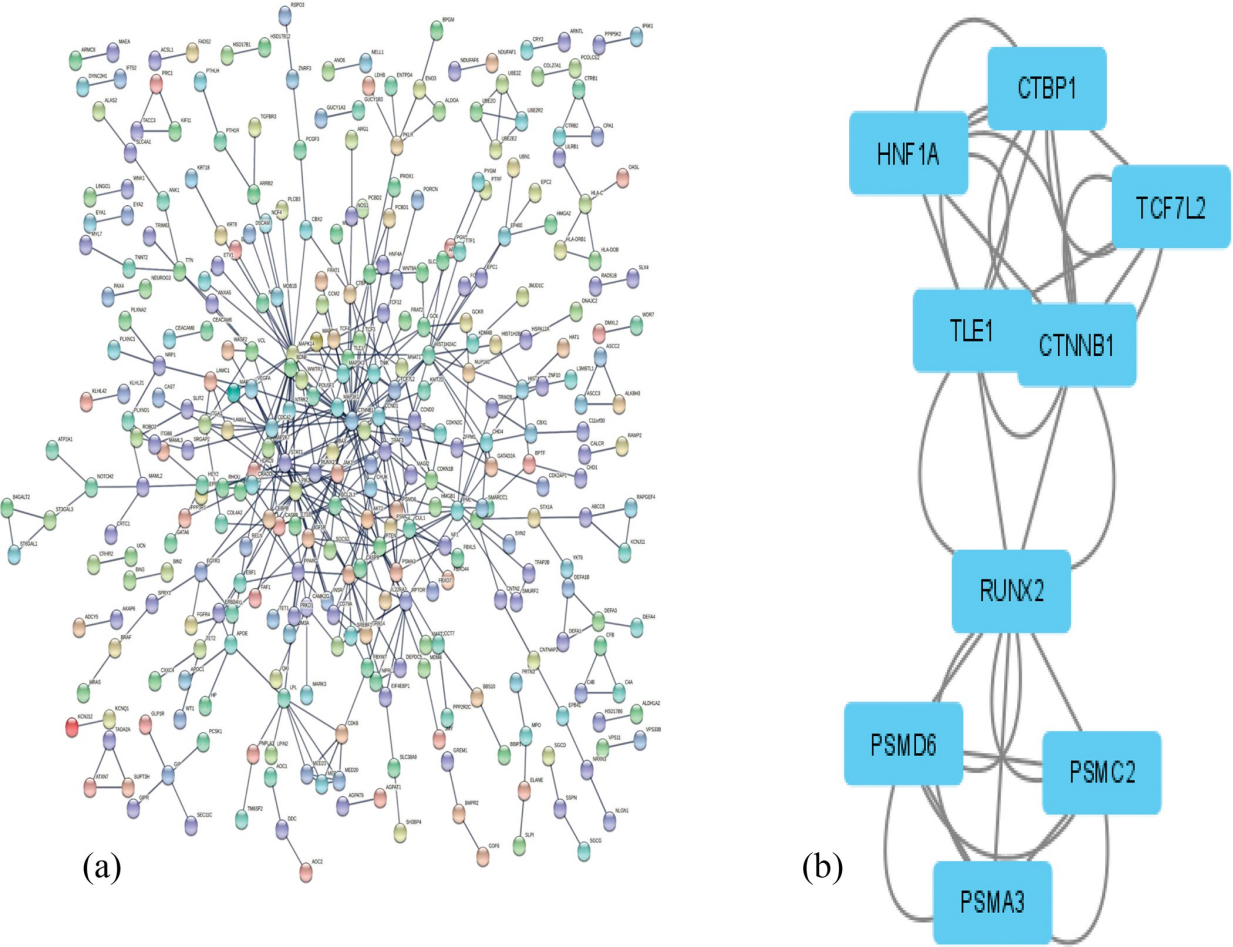
Protein-protein interaction network (a) obtained from the STRING database for (867) genes (interaction score > 0.9). The identified hub genes and their interactions (b). Circles represent genes, and lines represent interactions among proteins of differentially expressed genes.

As mentioned above, 249 and 843 DEGs from the GEO microarray and GWAS catalog datasets were associated with T2D pathogenesis in SAP respectively, that totaled 1092 DEGs. Then, overlapped genes were counted once, and therefore, 1085 unique genes were detected. Following that, the STRING database was used to construct PPI among 1085 proteins, and only 867 proteins with a confidence score of 0.9 were observed and presented in the PPI network (Fig 1A). Further, nine genes (*HNF1A*, *CTNNB1*, *PSMC2*, *PSMA3*, *RUNX2*, *TCF7L2*, *TLE1*, *PSMD6*, and *CTBP1*) were identified as hub genes following MCODE analysis (Fig 1B).

### Gene ontology enrichment analysis

**Fig 2:**
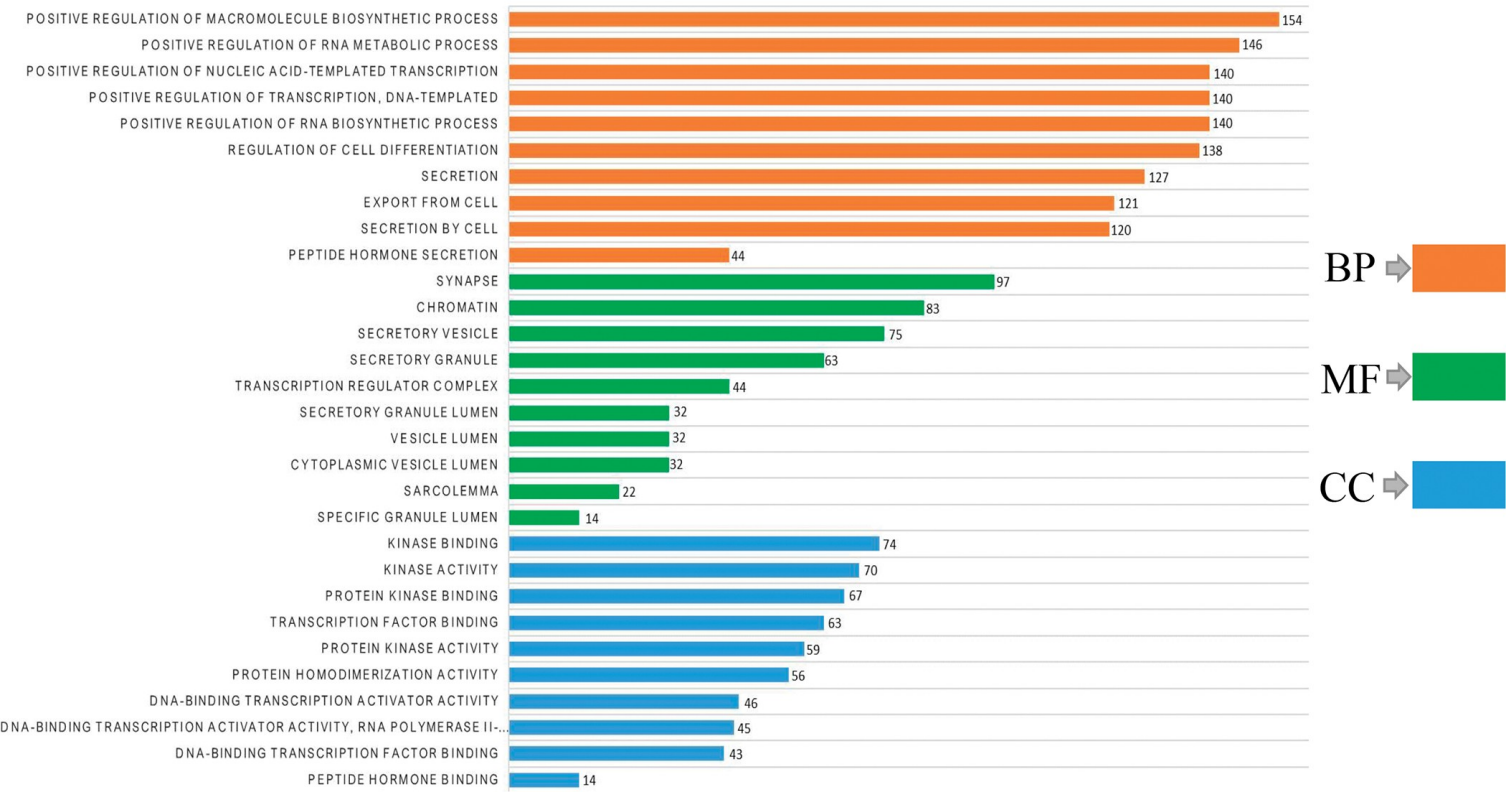
**The gene ontology enrichment analysis of differentially expressed genes.** The orange color represents the biological processes; the green color represents the cellular components and the blue color represents the molecular functions of candidate genes.

Gene ontology (GO) is a framework to classify how gene plays a role in molecular functions, biological processes, and cellular components. GO analysis of all 867 potential genes was performed using the ToppGene database. The top 10 significant molecular activities, biological processes, and cellular components were selected (Fig 2). Changes in biological processes were significantly enriched due to positive regulation of RNA metabolic processes, macromolecule biosynthetic processes, transcription, nucleic acid-templated transcription, DNA- and RNA-templated biosynthetic processes, cellular secretion, peptide hormone secretion, regulation of cell differentiation, and homeostasis of the cell (S3 Table). The cellular component analysis of DEGs revealed that these genes play a significant role in the vesicle lumen, granule lumen, transcription regulator complex, secretory granule lumen, sarcolemma, cytoplasmic vesicle lumen, secretory vesicle, chromatin, secretory granule, and synapse (S3 Table). Furthermore, based on the molecular functional analysis, the candidate genes significantly contributed to transcription factor binding, peptide hormone binding, DNA-binding transcription factor binding, kinase binding, DNA-binding transcription activator activity, kinase activity, protein kinase activity, protein kinase binding, DNA-binding transcription activator activity, protein homodimerization activity, and RNA polymerase II-specific activity (S3 Table).

### KEGG pathway enrichment analysis

The analysis of KEGG pathway enrichment based on the highest enrichment ratio and an FDR of < 0.05 revealed that DEGs regulate several metabolic pathways (S1 Fig). In the analysis, the most enriched pathways were maturity-onset diabetes of the young (MODY), EGFR tyrosine kinase inhibitor resistance, pancreatic cancer, insulin secretion, small cell lung cancer, prostate cancer, transcriptional misregulation in cancer, HIF-1 (Hypoxia-inducible factor 1) signaling pathway, the PI3K-Akt (Phosphoinositide 3-kinases-protein kinase B) signaling pathway, and human papillomavirus infection (Table 1).

**Table 1:** KEGG pathway analysis.

Pathway ID	Pathway Name	Ratio of enrichment	P-Value	FDR
hsa04950	Maturity onset diabetes of the young	6.8208	0.000002932	0.00047792
hsa01521	EGFR tyrosine kinase inhibitor resistance	3.9908	0.000001601	0.00047792
hsa05212	Pancreatic cancer	3.4155	0.000086209	0.0039929
hsa04911	Insulin secretion	3.2455	0.000082768	0.0039929
hsa05222	Small cell lung cancer	3.2127	0.000052321	0.0034113
hsa05215	Prostate cancer	3.0471	0.000097986	0.0039929
hsa04066	HIF-1 signaling pathway	2.9557	0.00013958	0.005056
hsa05202	Transcriptional mis-regulation in cancer	2.3307	0.00017009	0.0055449
hsa05165	Human papillomavirus infection	2.0925	0.000017175	0.0018664
hsa04151	PI3K/AKT signaling pathway	2.0039	0.000043913	0.0034113

### Construction of DEGs-miRNAs regulatory network

**Fig 3:**
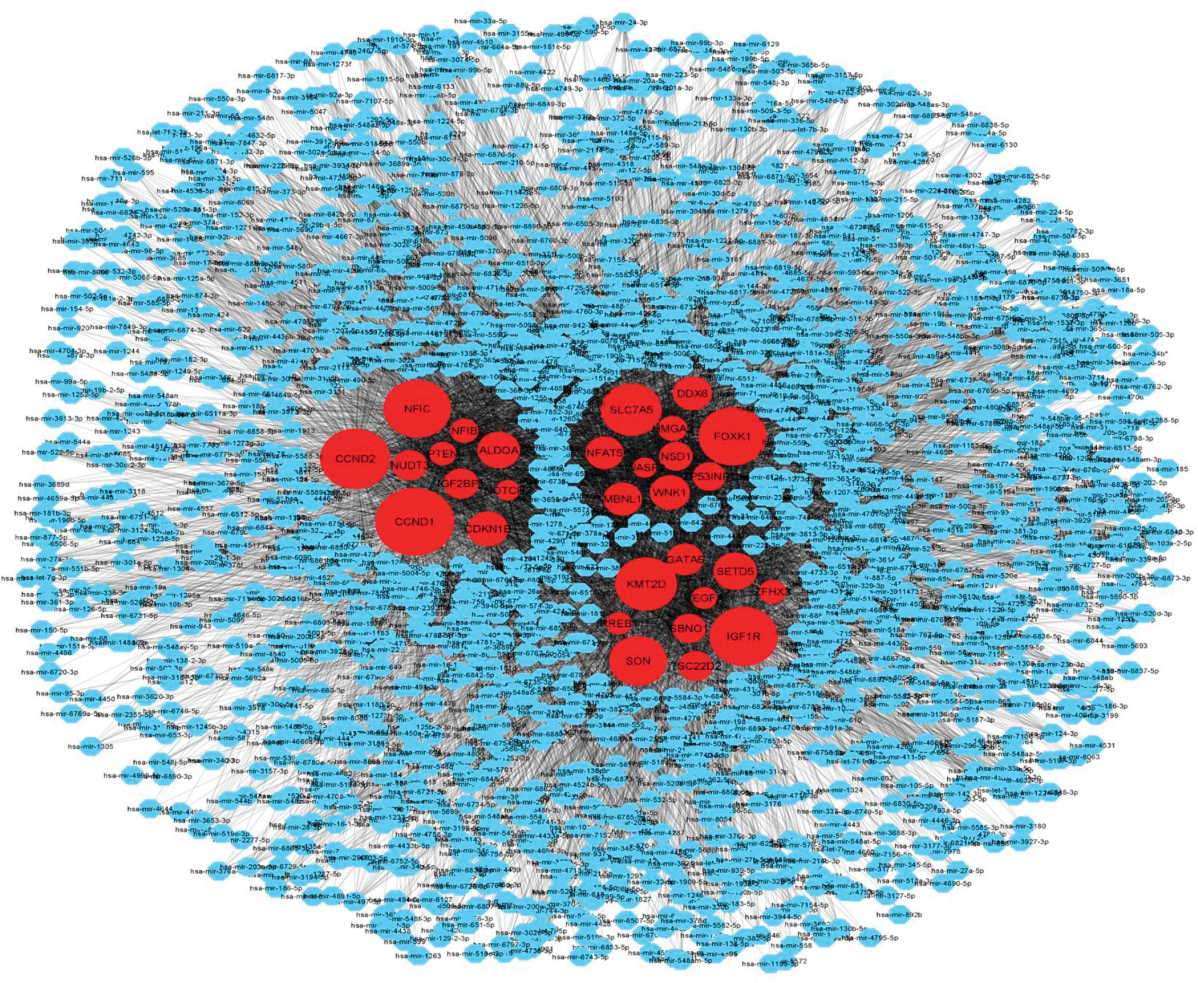
**Target gene–miRNA (microRNA) regulatory network between target genes and miRNAs.** The blue color diamond nodes represent the key miRNAs; Target genes are red colored.

The regulatory networks found that 825 DEGs were interrelated with 2582 miRNAs. Indeed, several miRNAs regulate the expression of a single gene (Fig 3). As observed, CCND1 was targeted by 396 miRNAs (ex, hsa-mir-15a-5p), CCND2 was targeted by 365 miRNAs (ex, hsa-mir-15a-5p), IGF1R was targeted by 359 miRNAs (ex, hsa-mir-16-5p), FOXK1 was targeted by 357 miRNAs (ex, hsa-mir-15a-5p), NFIC was targeted by 355 miRNAs (ex, hsa-mir-15a-5p), KMT2D was targeted by 336 miRNAs (ex, hsa-mir-15a-5p), SLC7A5 was targeted by 331 miRNAs (ex, hsa-mir-15a-5p), SON was targeted by 321 miRNAs (ex, hsa-mir-16-5p), SETD5 was targeted by 270 miRNAs (ex, hsa-mir-16-5p), and ALDOA was targeted by 267 miRNAs (ex, hsa-mir-24-3p) (S4 Table).

### Disease association analysis

**Fig 4:**
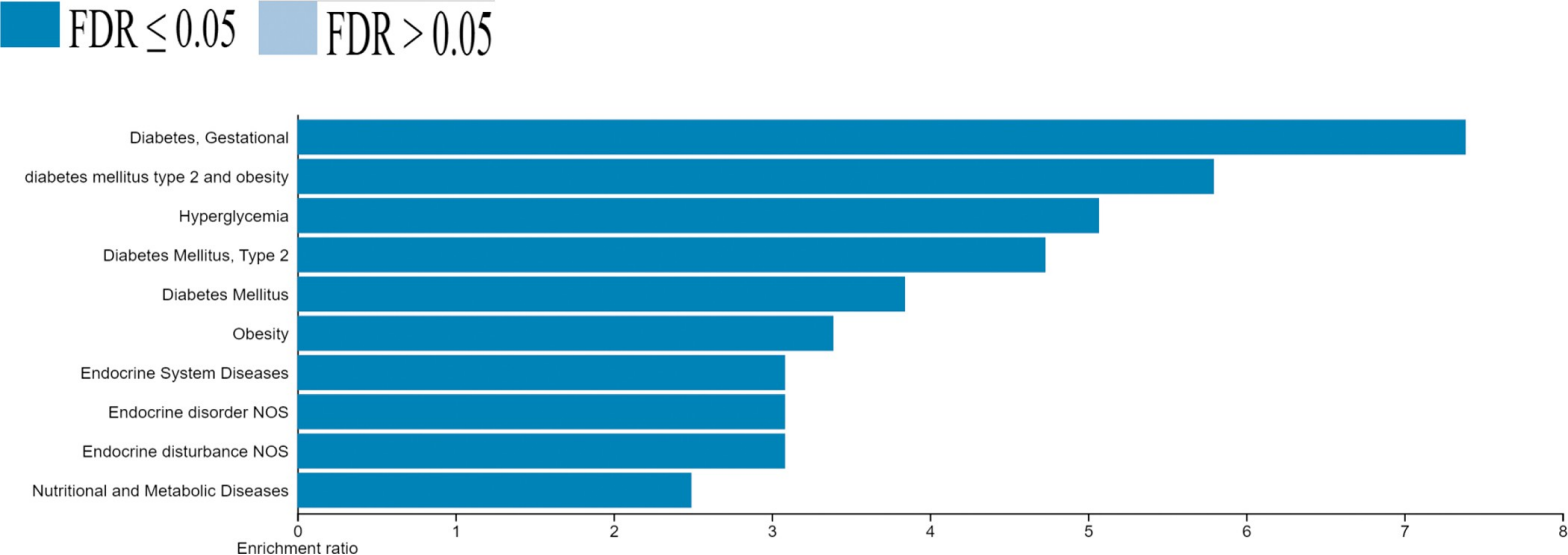
Disease association analysis of differentially expressed genes for type 2 diabetes. The associations are based on FDR values and enrichment ratios.

The disease association analysis was performed to identify the diseases associated with identified DEGs. The results revealed that gestational diabetes, T2D, obesity, hyperglycemia, endocrine system diseases, endocrine disorder NOS (Not Otherwise Specified), endocrine disturbance NOS, and nutritional and metabolic diseases are associated with identified DEGs (Fig 4). Among 867 genes, only 118 genes were significantly associated with T2D pathogenesis (Table 2). Two (HNF1A and TCF7L2), of 118 genes were identified in the hub genes network (Fig 2B), indicating these two genes have a significant role in T2D pathogenesis and its associated disorders.

**Table 2:** Disease association analysis.

Disease	Statistics	Genes
Diabetes, Gestational	C = 86; O = 32; E = 4.33; R = 7.39; raw. P = 0.000e+0; adj. P = 0.000e+0	ABCC8, ADRB3, CDC123, CDKAL1, FTO, GCK, GCKR, GIP, HHEX, HLA-DQB1, HNF1A, HNF1B, HNF4A, IGF1R, IGF2BP2, INSR, ITLN1, JAZF1, KCNJ11, KCNQ1, LEPR, MTNR1B, NEGR1, NUCB2, PPARG, SHBG, SLC30A8, TCF7L2, THADA, TMEM18, TSPAN8, WFS1
Diabetes mellitus type 2 and obesity	C = 161; O = 47; E = 8.11; R = 5.80; raw. P = 0.000e+0; adj. P = 0.000e+0	ADRB3, ARNTL, BDNF, C2CD4B, CDC123, CDKAL1, CRY2, FAIM2, FFAR2, FTO, GCK, GCKR, GIP, GIPR, GLP1R, HHEX, HNF1A, HNF1B, IGF2BP2, INSR, IRX3, ITLN1, JAZF1, KCNJ11, LEPR, LPL, MC4R, MTNR1B, NEGR1, NEUROG3, NOTCH2, PCSK1, PNPLA3, PPARG, SEC16B, SGIP1, SLC16A11, SLC2A2, SLC30A8, SPX, SREBF1, TCF7L2, THADA, TMEM18, TSPAN8, WFS1, ZNF654
Hyperglycemia	C = 141; O = 36; E = 7.10; R = 5.07; raw. P = 2.220e-16; adj. P = 6.671e-14	ABCC8, ADRB3, C2CD4B, CDKAL1, DGAT2, DGKB, FTO, GCK, GCKR, GIP, GIPR, GLP1R, HHEX, HNF1A, HNF1B, HNF4A, IGF1R, IGF2BP2, INSR, JAZF1, KCNJ11, LEPR, MTNR1B, NEUROG3, NOTCH2, PCSK1, PPARG, PROX1, SHBG, SLC2A2, SLC30A8, SREBF1, TCF7L2, THADA, TSPAN8, WFS1
Diabetes Mellitus, Type 2	C = 298; O = 71; E = 15.01; R = 4.73; raw. P = 0.000e+0; adj. P = 0.000e+0	ABCC8, ACE, ADCY5, ADRB3, AP3S2, APOE, ARAP1, ARL15, C2CD4B, CDC123, CDKAL1, CRY2, DGKB, FADS2, FAIM2, FTO, GCK, GCKR, GIP, GIPR, GLIS3, GLP1R, GRB14, HHEX, HMG20A, HNF1A, HNF1B, HNF4A, HP, IGF2BP2, INSR, IRX3, ITLN1, JAZF1, KCNJ11, KCNK16, KCNQ1, KIF11, KLF14, LEPR, LPL, MC4R, MTNR1B, NEGR1, NEUROG3, NOTCH2, NUCB2, PAX4, PCSK1, PEPD, PNPLA3, PPARG, PROX1, PTPRD, SEC16B, SHBG, SLC16A11, SLC22A2, SLC2A2, SLC30A8, SREBF1, TCF7L2, TFAP2B, THADA, TMEM154, TMEM18, TSPAN8, UBE2E2, VEGFA, VPS26A, WFS1
Diabetes Mellitus	C = 372; O = 72; E = 18.73; R = 3.84; raw. P = 0.000e+0; adj. P = 0.000e+0	ABCC8, ACE, ADCY5, ADRB3, AP3S2, APOE, ARAP1, ARL15, C2CD4B, CDC123, CDKAL1, CRY2, DGKB, EPO, FADS2, FTO, GCK, GCKR, GIP, GIPR, GLIS3, GLP1R, GRB14, GREM1, HHEX, HLA-DQB1, HLA-DRB1, HNF1A, HNF1B, HNF4A, HP, IGF2BP2, INSR, IRX3, ITLN1, JAZF1, KCNJ11, KCNK16, KCNQ1, KL, KLF14, LEPR, LPL, MC4R, MTNR1B, NEGR1, NEUROG3, NOTCH2, NUCB2, OLR1, PAX4, PCSK1, PNPLA3, PPARG, PROX1, PTPRD, RASGRP1, SHBG, SLC16A11, SLC22A2, SLC2A2, SLC30A8, SREBF1, TCF7L2, THADA, TMEM154, TMEM18, TSPAN8, UBE2E2, VEGFA, VPS26A, WFS1
Obesity	C = 369; O = 63; E = 18.58; R = 3.39; raw. P = 0.000e+0; adj. P = 0.000e+0	ACE, ADRB1, ADRB3, APOC1, APOE, ARNTL, BBIP1, BBS10, BDNF, C5orf67, CDC123, CDKAL1, CENPW, CRY2, DGAT2, EBF1, FADS2, FAIM2, FFAR2, FTO, GCK, GCKR, GIP, GIPR, GLP1R, GRB14, HHEX, HP, IGF2BP2, INSR, IRX3, ITLN1, JAZF1, KCNJ11, LEPR, LPIN2, LPL, MAP2K5, MC4R, MGAT1, MSRA, MTNR1B, NEGR1, NRXN3, NUCB2, PCSK1, PNPLA3, POC5, PPARG, SEC16B, SHBG, SLC16A11, SLC30A8, SLC39A8, SPX, SREBF1, TCF7L2, TFAP2B, THADA, TM6SF2, TMEM18, TRIM66, TSPAN8
Endocrine System Diseases	C = 457; O = 71; E = 23.01; R = 3.09; raw. P = 0.000e+0; adj. P = 0.000e+0	ABCC8, ACE, ACVR1C, ADCY5, ADRB3, AKT2, ARAP1, BRAF, C2CD4B, CASR, CDC123, CDKAL1, CDKN1B, CDKN2C, DGKB, FTO, GATA6, GCK, GCKR, GIP, GIPR, GLIS3, GLP1R, HHEX, HLA-DQB1, HLA-DRB1, HMGA2, HNF1A, HNF1B, HNF4A, HP, IGF1R, IGF2BP2, INSR, ITLN1, JAZF1, KCNJ11, KCNK16, KCNQ1, KL, KLF14, KRT19, LEPR, LPL, MC4R, MTNR1B, NEGR1, NEUROG3, NOTCH2, NUCB2, PAX4, PCSK1, PIM3, PPARG, PROX1, PTEN, RREB1, SHBG, SLC16A11, SLC2A2, SLC30A8, TCF7L2, TG, TGFBR3, THADA, TMEM18, TSPAN8, VEGFA, WFS1, WT1, ZFAT
Endocrine disorder NOS	C = 457; O = 71; E = 23.01; R = 3.09; raw. P = 0.000e+0; adj. P = 0.000e+0	ABCC8, ACE, ACVR1C, ADCY5, ADRB3, AKT2, ARAP1, BRAF, C2CD4B, CASR, CDC123, CDKAL1, CDKN1B, CDKN2C, DGKB, FTO, GATA6, GCK, GCKR, GIP, GIPR, GLIS3, GLP1R, HHEX, HLA-DQB1, HLA-DRB1, HMGA2, HNF1A, HNF1B, HNF4A, HP, GF1R, IGF2BP2, INSR, ITLN1, JAZF1, KCNJ11, KCNK16, KCNQ1, KL, KLF14, KRT19, LEPR, LPL, MC4R, MTNR1B, NEGR1, NEUROG3, NOTCH2, NUCB2, PAX4, PCSK1, PIM3, PPARG, PROX1, PTEN, RREB1, SHBG, SLC16A11, SLC2A2, SLC30A8, TCF7L2, TG, TGFBR3, THADA, TMEM18, TSPAN8, VEGFA, WFS1, WT1, ZFAT
Endocrine disturbance NOS	C = 457; O = 71; E = 23.01; R = 3.09; raw. P = 0.000e+0; adj. P = 0.000e+0	ABCC8, ACE, ACVR1C, ADCY5, ADRB3, AKT2, ARAP1, BRAF, C2CD4B, CASR, CDC123, CDKAL1, CDKN1B, CDKN2C, DGKB, FTO, GATA6, GCK, GCKR, GIP, GIPR, GLIS3, GLP1R, HHEX, HLA-DQB1, HLA-DRB1, HMGA2, HNF1A, HNF1B, HNF4A, HP, IGF1R, IGF2BP2, INSR, ITLN1, JAZF1, KCNJ11, KCNK16, KCNQ1, KL, KLF14, KRT19, LEPR, LPL, MC4R, MTNR1B, NEGR1, NEUROG3, NOTCH2, NUCB2, PAX4, PCSK1, PIM3, PPARG, PROX1, PTEN, RREB1, SHBG, SLC16A11, SLC2A2, SLC30A8, TCF7L2, TG, TGFBR3, THADA, TMEM18, TSPAN8, VEGFA, WFS1, WT1, ZFAT
Nutritional and Metabolic Diseases	C = 709; O = 89; E = 35.71; R = 2.49; raw. P = 6.661e-16; adj. P = 1.801e-13	ABCC8, ACE, ADCY5, ADRB3, ALAS2, ANKH, APOC1, APOE, ARAP1, ARL15, BTD, C2CD4B, C5orf67, CALCR, CASR, CDC123, CDKAL1, DGAT2, DGKB, FADS2, FAIM2, FTO, FXYD2, GALNT3, GCDH, GCK, GCKR, GIP, GIPR, GLIS3, GLP1R, GRB14, GRN, HHEX, HLA-DQB1, HLA-DRB1, HMBS, HNF1A, HNF1B, HNF4A, HP, IGF2BP2, INSR, IRX3, ITLN1, JAZF1, KCNJ11, KCNK16, KCNQ1, KL, KLF14, LEPR, LPIN2, LPL, MC4R, MMAA, MTNR1B, NDUFAF6, NEGR1, NEUROG3, NOTCH2, NPC2, NUCB2, OLR1, PAX4, PCSK1, PGM1, PKLR, PNPLA3, PPARG, PYGM, SEC16B, SHBG, SLC16A11, SLC2A2, SLC30A8, SLC34A1, SLC4A1, SLX4, SPX, SREBF1, TCF7L2, TFAP2B, TFRC, THADA, TMEM106B, TMEM18, TSPAN8, WFS1

**The statistic column lists:** C- the number of reference genes in the category; O- the number of genes in the gene set and in the category; E- the expected number in the category; R- the ratio of enrichment; raw P- p-value from hypergeometric test; adj. P- p-value adjusted by the multiple test adjustment.

### Construction of the drug-gene network

**Fig 5:**
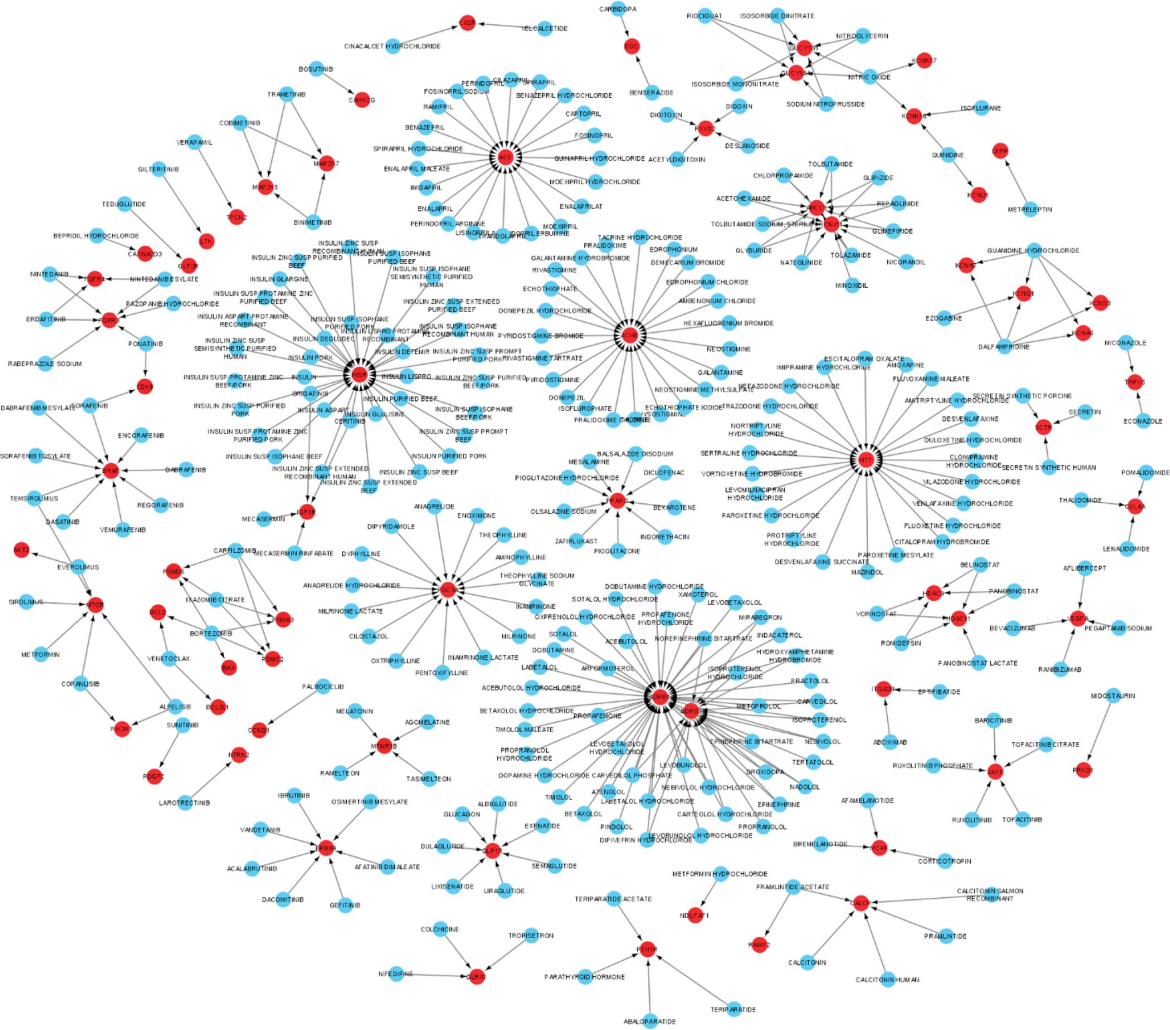
Drug-gene interaction network. Red dot indicates the genes and blue dot indicates drugs known to inhibit the expression of target genes.

Research on drug-gene interaction networks is crucial for drug discovery and development. Here, the networks of candidate drug-gene were built based on the interactions and effects of the medications. The candidate drug-gene interactions were constructed using 867 DEGs obtained from the PPI network, that may guide to explore the mechanism for treating T2D (Fig 5). In drug-gene networks, 64 genes were interacted with 367 drugs of T2D. Among the 64 genes, 11 genes (ABCC8, ACE, ACHE, ADRB1, ADRB3, BRAF, HTT, INSR, KCNJ11, PDE3A, and PPARG) were downregulated by 10, 21, 23, 43, 18, 9, 23, 34, 13, 15, and 9 drugs, respectively, that are FDA-approved (S5 Table).

## Discussion

Comprehensive analysis of the microarray dataset guides expression patterns of DEGs and their integrative biological functions under different conditions in living organisms [31]. To identify and characterize DEGs, the raw data should be analyzed after omitting poor-quality measurements, simplifying comparisons, fixing measured intensities, and proper screening [33–35]. Following that, normalization is usually done to identify significant biological associations of the expression data. Analysis of gene expression under different conditions guides how the gene plays a role in different biological functions [36].

Here, we have screened 1085 DEGs from microarray (GEO) and GWAS catalog datasets after removing overlapped genes. Microarray dataset analysis revealed that 249 genes were associated with T2D pathogenesis, where 121 were upregulated and 128 downregulated (S1 Table). More specifically, PTGS2 and IL1B genes were upregulated in children with diabetes [37]. A number of 29 genes were upregulated, and two were downregulated in patients having acute hyperinsulinemia in skeletal muscle [38]. In addition, 109 upregulated and six downregulated genes were observed in T2D [22]. Interestingly, 301 upregulated and 680 downregulated genes between T2D and the control population were also observed [39]. More specifically, the ABRA gene is upregulated when skeletal muscle is insulin-resistant [40]. Here, we have also identified 843 unique genes from 17 GWAS catalog datasets that are associated with T2D pathogenesis in South Asian populations (S2 Table). Accordingly, 233 unique genes from the GWAS catalog data were found to be associated with T2D [22]. Hence, DEGs play critical roles in T2D pathogenesis, which may be pivotal in treating diabetes by investigating their regulatory functions [39,41]. This current study is the first comprehensive analysis of the GEO and GWAS catalog datasets among the SAPs and identifies potential DEGs related to T2D.

In the PPI network, we have identified 867 candidate genes that are significantly associated with T2D pathogenesis. In addition, we have found nine hub genes among the 867 candidate genes for T2D pathogenesis. Among the nine hub genes, two genes (CTNNB1, RUNX2) were only found in the South Asian population. However, the remaining seven hub genes (HNF1A, PSMC2, PSMA3, TCF7L2, TLE1, PSMD6, CTBP1) were observed in the South Asian population in addition to other major populations (American, African, East Asian, and European).

Further, the integrated results of module selection (Fig 1) and disease association analysis (Fig 4) of 867 DEGs identified HNF1A and TCF7L2 genes play a crucial role in T2D pathogenesis and its related diseases. The pathway analysis showed that HNF1A and TCF7L2 genes were responsible for maturity-onset diabetes of the young (MODY) and human papillomavirus infection and prostate cancer pathways, respectively. Indeed, TCF7L2 [42,43] and HNF1A genes play a significant role in T2D pathogenesis [44]. Meta-analysis in a multi-ancestry among 1.4 million participants revealed that TCF7L2 (rs35011184-G) and HNF1A (rs56348580-G) increased the risk of T2D pathogenesis [45]. Saxena et al. (2013) indicated that TCF7L2 (rs7903146-T) increases the risk of T2D in the SAP [46]. Our previous study also found that 22 candidate genes significantly contribute to T2D pathogenesis among Asian populations [47].

The results of GO analysis demonstrated that most biological processes were linked to regulating RNA and DNA-based metabolic and biosynthetic processes. Furthermore, most cellular components were found to be linked with granule and vesicle lumen activities. The result is consistent with our previous research except for differences in population group [47]. By binding with homologous DNA and RNA, lncRNAs control gene expression and are linked to various human diseases, including diabetes [48]. Hence, the crucial roles of the analyzed DEGs in different biological systems guide how DEGs play a role in T2D pathogenesis modulating cellular, molecular and biological processes in SAP.

DEGs are observed in the secretory-granule lumen, vesicle lumen, and platelet alpha granule lumen tissues in T2D [49]. Insulin is secreted in the vesicles called insulin secretory granules (ISGs) that are affected by T2D, consequences of dysfunctional ISG production, and restricted insulin secretion [50]. In our analysis, most molecular functions of the identified DEGs were linked to transcription factors and kinase-binding activities (S3 Table).

MODY is a genetic heritable diabetes characterized by beta-cell dysfunction, non-insulin-dependent diabetes (NIDD), and autosomal dominant inheritance at a young age [51]. It is also known as non-ketotic diabetes, caused due to malfunctioning of pancreatic beta-cell, and lack of pancreatic autoantibodies [52]. The epidermal growth factor receptor (EGFR), a tyrosine kinase receptor having a transmembrane domain, is the critical component of cell signaling pathways. The EGFR receptor plays a vital role in the MAPK (Mitogen-activated protein kinase) pathway, the PI3K/AKT (Phosphatidylinositol-3-kinase/Protein kinase B) pathway, and the JAK (Janus kinase) pathway, which stimulates cell proliferation, mitosis, and inhibition of apoptosis [53]. The heterodimeric transcription factor HIF-1 is the critical mediator that controls the expression of numerous genes involved in cell cycle regulation, cellular metabolism, angiogenesis, and block of apoptosis [54]. In addition, the PI3K/AKT pathway regulates many cellular functions, like cell survival, cancer progression, proliferation, neuroscience, and metabolism [55]. In the study, the pathway enrichment analysis showed that the identified DEGs associated with T2D significantly interacted with the MODY, EGFR, PI3K-AKT, and HIF-1 signaling pathways and, therefore, might play a significant regulatory network in the progression of T2D pathogenesis (Table 1 and S1 Fig).

miRNA plays a regulatory role in disease progression through epigenetic modification, histone modification, and DNA methylation. miRNAs are also associated with the diagnosis and response for the treatment of diseases [56]. miRNAs are commonly found in all human/mammal cells that are involved in cell development [57] by regulating around 30 percent of the genes that code proteins [58]. miRNAs critically regulate post-transcriptional gene expression [59]. miRNAs are also involved in glucose homeostasis and regulate the expression of genes involved in diabetes-relevant pathways like the insulin signaling pathway [60–62]. Some miRNAs also control the secretion and synthesis of insulin to balance blood glucose levels in human [63]. miR-362-3p, miR-15a-5p, miR-150-5p, and miR-877-3p showed significant contributions in T2D pathogenesis [64]. Transcripts of hsa-miR-16-5p, hsa-miR-17-5p, hsa-miR-19a-3p, and hsa-miR-20a-5p miRNAs were upregulated in patients having insulin resistance and abnormal pregnancies. Gene-miRNAs regulatory network analysis revealed that these miRNAs significantly regulate MAPK signaling, insulin signaling, TGF-β signaling, and mTOR signaling pathways consequences the progression of T2D [65]. Increased expression of has-miR-24-3p plays a crucial role in the pathophysiology and progresses of proliferative diabetic retinopathy [66]. Increased expression of has-miR-15a-5p stimulates β cells and promotes insulin production [62]. Overexpression of has-miR-27a-3p in L6 cells decreased glucose consumption and glucose uptake and reduced the expression of GLUT4, MAPK 14, and PI3K regulatory subunit [67]. miRNA hsa-let-7a-5p was shown to be significantly associated with diabetic retinopathy (DR) in T2D. Overexpression of hsa-let-7a-5p resulted in rapid pathogenesis of DR [68]. In our study, we have identified hsa-mir-16-5p, hsa-mir-17-5p, hsa-mir-24-3p, hsa-mir-27a-3p, hsa-let-7a-5p, hsa-mir-19a-3p, hsa-mir-15a-5p, and hsa-mir-20a-5p that are significantly interacted with the identified DEGs associated with T2D (S4 Table). Therefore, the interacted miRNAs may cause progression of T2D pathogenies regulating MAPK signaling, insulin signaling, TGF-β signaling, mTOR signaling pathways, and diabetic retinopathy signaling pathways.

Furthermore, 367 FDA-approved drugs for T2D significantly downregulated the candidate genes in drug-gene association analysis (S5 Table). D-phenylalanine derivative nateglinide is an amino acid that stimulates insulin secretion by regulating pancreatic β-cells. It also controls hyperglycemia and improves glycemic control in T2D [69]. The another drug carvedilol helps to improve endothelial functions in T2D [70]. Interestingly, the T2D drug diclofenac sodium plays a significant role in glycemic control [71], and dipyridamole significantly reduces proteinuria in T2D nephropathy [72]. In addition, repaglinide is an efficient anti-diabetic drug [73]. In our study, nateglinide significantly downregulated the expression of ABCC8, and KCNJ11 genes (S5 Table). In addition, repaglinide significantly downregulated the expression of ABCC8, and KCNJ11 genes, carvedilol downregulated ADRB1, and ADRB3 genes (S5 Table). Indeed, diclofenac and dipyridamole significantly downregulated expression of PPARG, and PDE3A genes respectively (S5 Table). The case may same for other drugs. Therefore, in addition to above mentioned drugs, other drugs found in the gene-drug interaction network could be used to downregulate the expression of candidate DEGs for treating T2D. Since different drugs interacted with specific genes, the precise drug might be recommended to specific T2D patients after observing genetic mutations, and expression levels of candidate genes for curing T2D, even controlling of progression of prediabetes to T2D [74].

## Conclusions

Since genetic signatures play vital roles in T2D pathogenesis, a series of bioinformatic systems were applied to analyze 2 GEO and 17 GWAS catalogue datasets from SAPs to explore the DEGs associated with the diseases. Following critical PPI analysis, 867 DEGs were found to be associated with T2D pathogenesis. Indeed, nine hub genes were also identified for the pathogenesis. Among these, CTNNB1, and RUNX2 could be the markers for T2D pathogenesis in SAPs as only found in that populations. In GO analysis, most of the identified DEGs showed significant contributions in molecular activities, biological processes, and cellular components of T2D. Following KEGG pathway analysis, MODY, EGFR tyrosine kinase inhibitor resistance, insulin secretion, HIF-1, and PI3K-Akt signaling pathways were found to be significantly enriched by the DEGs. Two genes (HNF1A and TCF7L2) among 118 identified genes that significantly contributed to T2D, were found in both hub genes and disease association. Even, 825 DEGs were also interrelated with 2582 miRNAs. Not surprisingly, several miRNAs regulate the expression of a single gene or vice versa (ex, hsa-mir-15a-5p, hsa-mir-16-5p, hsa-mir-24-3p). Among the 64 genes that interacted with 367 drugs of T2D, ABCC8, ACE, ACHE, ADRB1, ADRB3, BRAF, HTT, INSR, KCNJ11, PDE3A, and PPARG genes were downregulated by wide range of (9–43) FDA approved drugs for T2D. Indeed, different FDA-approved drugs significantly downregulated the expression of target genes. Therefore, the findings of the research might guide to explore the mechanism of how the DEGs progress T2D pathogenesis by interacting different biological functions, pathways, and miRNAs. Considering the above-mentioned findings, precise medication could be recommended after diagnosing the molecular mechanism of T2D pathogenesis and observing the expression levels of marker genes in T2D patients among SAPs.

## Supporting information

S1 Fig KEGG pathway enrichment analysis. The pathway is based on the FDR value and enrichment ratio.
(DOCX)

S1 Table Differentially expressed genes identified from microarray analysis. (DOCX)

S2 Table Genome-wide association study (GWAS). (DOCX)

S3 Table The top 10 most significant gene ontology (GO) terms. (DOCX)

S4 Table Target gene—miRNA regulatory networks. (DOCX)

S5 Table Drug-gene interaction networks. (DOCX)
