# Supplementary material for: In silico identification and functional prediction of differentially expressed genes in South Asian populations associated with type 2 diabetes
Source: PLoS One. 2023 Dec 14;18(12):e0294399. doi: 10.1371/journal.pone.0294399 (PMC10721103; doi:10.1371/journal.pone.0294399)

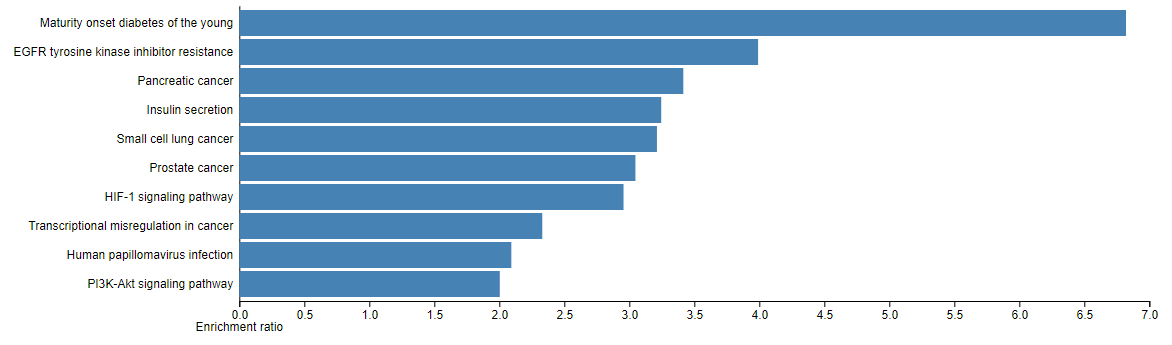


**S1 Fig.** KEGG pathway enrichment analysis. The pathway is based on the FDR (false discovery rate) value and enrichment ratio.


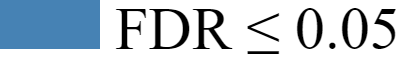

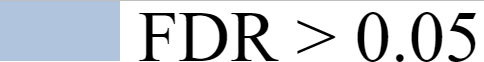

Supplement: S1 Fig — The pathway is based on the FDR value and enrichment ratio. (DOCX) [file pone.0294399.s001.docx]
