# Supplementary material for: In silico identification and functional prediction of differentially expressed genes in South Asian populations associated with type 2 diabetes
Source: PLoS One. 2023 Dec 14;18(12):e0294399. doi: 10.1371/journal.pone.0294399 (PMC10721103; doi:10.1371/journal.pone.0294399)
Supplement: S1 Table — (DOCX) [file pone.0294399.s002.DOCX]

| **S1 Table.** Differentially expressed genes identified from microarray analysis | | | | | |
| --- | --- | --- | --- | --- | --- |
| **GSE_ID** | **Country** | **p-value** | **Fold change value** | **Up-regulated** | **Down-regulated** |
| GSE26168 | Singapore | p-value < 0.05 | \|logFC\| > 1.5 | DEFA1B, HBZ, DEFA3, NPRL3, SON, RBM38, OLFM4, DEFA1, WNK1, PLXNC1, HP, CEACAM8, SLC4A1, KRT1, BCL2L1, LTF, DNASE1L1, MPO, CEACAM6, CASP8, PPM1A, ARID4B, VCL, CDK8, PRTN3, BIN2, FBXO7, DEFA4, DNAJB12, MICALCL, BCAS4, EPB41, ITGA2B, LIMS1, CDC42, CNTNAP2, IL19, ARMT1, CNN2, FFAR2, AGBL5, TADA2A, RTN3, WASF2, BPGM, LTBP3, HM13, FAM101B, GRN, AMD1, NCF4, CCNB1IP1, IL1RN, CDC14B, BPI, ZFYVE1, FBXL5, FAM134B, BABAM1, JAK1, HIST1H2AC, CCT7, TRIM28, PCOLCE2, ALDOA, NFYA, ST3GAL3, ELANE, CEP290, ARMC8, EPC1, MICAL2, STX1A, GUCY1A3, GPHB5, ENTPD4, MFHAS1, CTNNB1, NDST1, TSC22D1, ALAS2, TRIM5, PCNX2, OR2W3, LYPLA2, ATP5F1, PPP3R1, TRIM58, DMXL2, C21ORF33, CCM2, OLR1, TMCC2, CR2, DDX6 | DYNC2H1, TNNT2, CD79A, ASCC3, RHOT1, ZHX3, C9orf131, CBX2, EFNA4, DSCAM, SH3BP4, LTK, PTPRG, KIAA1033, ZNF326, IRX1, RBBP9, MAP3K12, MAPK14, ARRB2, CD1E, PLXNA2, KCTD7, BBS10, POLG2, FLJ26850, ICE2, ARHGAP5, NDUFAF1, CASP9, AOC2, SERINC4, SMURF2, MECOM, MLLT3, ITM2B, KMT2D, FAM171B, FGFR3, IGSF8, FBXO42, CRMP1, ARHGAP36, RAPGEF4, PRSS42, FRMD6, IP6K1, SGK2, KCNA6, GALNT8, ADGRA3, RAD51B, ZNF485, FAM177A1, ZNF580, TRPS1, MIER2, GSG1L, EHD3, CHST2, OR56B1, UBN1, FBXO44, PORCN, A4GNT, AKT2, SLC34A1, COL4A2, ADRB3, MAST2, UBE2R2, MSR1, FAM151B, GPAT4, HDAC11, PYGM, SCN4B, PRSS21, LRRK1, NFAM1, STAC3, ERLIN1, MAGED2, SLCO3A1, UCN, ARHGEF10L, SPECC1, SH3BGR, NRP1, TTF1, AIFM3, CBARP, RUNX2, ZNF579, CUL4A, MPV17L, 12-Sep, ADPRH, PIK3R1, IL22RA2, SENP7, EPHX1, EIF4EBP1, LETM1, CREB3, BAX, KLRC2, CCL20, C17orf47, SLC39A7, ABCC6, L3MBTL1, NFIC, B4GALT2, UFSP1, CCDC85B, ZSCAN25, XKR6, SYNPO, UXS1, CXXC4, MMAA, SLAMF8, CDC42, HLA-DRB1, QKI, GDF11 |
| GSE78721 | India |  |  | ITLN1, CLDN1, KRT18, ANXA8, ANXA8L1, LINC00842, C4A, C4B, C4B_2, GREM1, KRT19, BNC1, WT1, CFB, RARRES1, ITGB8, PTGDS, C7, IGHG1, IGHG3, IGHM, IGHV4-31, SLPI, KRT8, TFPI2, GATA6 | CRNDE, SPX |
