# Supplementary material for: In silico identification and functional prediction of differentially expressed genes in South Asian populations associated with type 2 diabetes
Source: PLoS One. 2023 Dec 14;18(12):e0294399. doi: 10.1371/journal.pone.0294399 (PMC10721103; doi:10.1371/journal.pone.0294399)
Supplement: S2 Table — (DOCX) [file pone.0294399.s003.DOCX]

| **S2 Table.** Genome-wide association study (GWAS) | |
| --- | --- |
| **GWAS Study accession** | **Gene** |
| GCST002352  (76) | LPP-AS2, LINC01991, CRHR2, PCBD2, TXNDC15, CDKAL1, COBLL1, GRB14, GLIS3, VPS33B, ANK1, NDUFAF6, UBE2E2, KCNJ11, RPS3AP18, TMEM154, KCNK16, FAF1, POU5F1, ARL15, MPHOSPH9, RN7SL836P, GIPR, TMEM18, RNF6, AP3S2, ETV1, IGF2BP2, PRELID1P1, RNU6-200P, RPL35AP3, ,LINC00824, TCF7L2, RPL5P26, LINC02651, MED6P1, PTEN, HHEX, Y_RNA, CDC123, RN7SL198P, ARAP1, SLC30A8, JAZF1, RNU4-17P, DGKB, SSR1, RREB1, ST6GAL1, MTNR1B, PROX1-AS1, HNF4A, PLEKHA1, RPSAP52, SPRY2, LINC01080, LINC02537, VEGFA, FTO, ZC3H11B, PPARG, HNF1B, DMRTA1, CDKN2B-AS1, HNF1A, PTHLH, RN7SKP15, NPM1P47, C2CD4B, MIR4432HG, WFS1, TLE1, RNU6-1035P, HMG20A, ZMIZ1, ADCY5 |
| GCST001213  (7) | COBLL1, GRB14, ST6GAL1, VPS26A, HMG20A, AP3S2, HNF4A |
| GCST008833  (24) | KCNQ1, SLC30A8, PPARG, GPSM1, HNF4A, SPRY2, LINC01080, KCNJ11, NTRK2,  TCF7L2, CDC123, CDKAL1, IGF2BP2, CTBP1-DT, RN7SL644P, EIF2S2P3, ASCL2, MIR4686, SFI1, ZMIZ1, JAZF1, COBLL1, CCND2, CCND2-AS1 |
| GCST004894  (111) | RPS3AP18, ARL15, C5orf67, ZBED3-AS1, JADE2, SSR1, CDKAL1, POU5F1, TSBP1-AS1, MIR588, DGKB, GTF3AP5, JAZF1, GCK, BRAF, ANK1, SLC30A8, CDKN2B-AS1, DMRTA1, CHCHD2P9, FAM234A, FTO, CTRB2, CMIP, HNF1B, RNU4-17P, MC4R, BCL2, GATAD2A, PEPD, APOC1P1, GIPR, HNF4A, SLC9B2, MACF1, FAF1, NOTCH2, PROX1, GCKR, THADA, MIR4432HG, MIR4435-2HG, COBLL1, GRB14, RN7SL198P, CDC123, ZMIZ1, HHEX, TCF7L2, PLEKHA1, MIR4686, KCNQ1OT1, KCNQ1, KCNJ11, ARAP1, MTNR1B, CCND2-AS1, PTHLH, RPSAP52, TSPAN8, OASL, ABCB9, RNF6, TOMM22P3, SPRY2, MIR17HG, C2CD4B, HMG20A, AP3S2, PRC1, KCNK16, SLC22A2, SMARCE1P4, NDUFAF6, GLIS3, TLE1, GPSM1, ITPR2, IGF2BP2, LINC01875, LINC01991, PPARG, UBE2E2, ADCY5, LPP-AS2, MAEA, WFS1, ANXA5, LINC02495, ADAMTS9-AS2, SMIM43, LINC01080, PPIAP23, NPM1P47, PRC1-AS1, TMEM154, RPL26P19, CENPW, SLC22A3, KCNU1, TP53INP1, RNU6-1035P, Y_RNA, SSPN, RN7SKP15, CTRB1, APOC1, PROX1-AS1, TMEM18, ASCL2, KL |
| GCST001033  (18) | MARCHF1, PCNX2, LPIN2, CDKAL1, DCDC2C, HMG20A, HUNK, KIF11, PEX5L, CR2, SDHAF4, FILNC1, ACHE, TCERG1L, RNU6-461P, ZPLD1, ACP7, PLS1 |
| GCST001759  (7) | TMEM163, CCNT2-AS1, MAP3K1, TGFBR3, TCF7L2, CDKN2B-AS1, FTO |
| GCST001809  (12) | TCF7L2, SGCG, SACS, SGCD, RHOU, CCN4, TG, NXN, MRM3, IGF2BP2, HMGB1P1, RBM38 |
| GCST005414  (33) | CTRB1, CTRB2, SPRY2, LINC01080, PROX1, PROX1-AS1, LINC02537, VEGFA, AP3S2, ARPIN-AP3S2, MIR6074, HMGA2, RPS3AP18, TMEM154, MIR4432HG, KLHL42, RN7SKP15, GTF3AP5, ADCY5, WFS1, PPP2R2C, SNRPGP16, MTNR1B, ZMIZ1, RN7SL836P, GIPR, ZC3H11B, ABO, CAMKK2, UBE2Z, ABCC8, GPAT4, NKX6-3 |
| GCST010557  (695) | MIRLET7A1HG, PTPDC1, FAIM2, AOC1, HIVEP2, SLC9B1, RMST, OR10AD1, H1-7, RMC1, NEUROD2, CDK12, INSR, SLCO4A1, NUP160, MIR924HG, BBIP1, PDZRN4, PML, TET2, STAG1, LINC01141, STEAP2-AS1, STEAP2, HDAC9, PTEN, GGNBP1, CRY2, SLC35C1, LRRC66, DCUN1D4, RGS17, AKAP6, TET1, HSD17B6, PRIM1, CLEC10A, SLC16A11, LINC01741, SEC16B, ACE, LCORL, CYTH1, DDX39AP1, SNORD36, RN7SKP114, DCAF12, KCNK16, KCNK17, USP3, USP3-AS1, COPB1, NF1, SCHLAP1, JMY, GNAS-AS1, PDGFC, LINC02272, TFRC, LINC00885, TRIM59, SREBF1, SLX4, RBM6, HORMAD2-AS1, SCD5, NFATC2, DTNB, PPP4R3A, EIF4E2P2, PHKG1P3, GTF2IP11, MEG3, OR4C9P, OR4R1P, MTOR, STK31, CDH7, SHQ1, RPL31P12, NEGR1, PLXND1, LINC00370, EHHADH-AS1, LMF1, LINC01829, ZFPM2, TBCE, LRRC74A, NHSL1, GCKR, TLE1, RNU6-1035P, GRB14, COBLL1, ZMIZ1, PPARG, FTO, IGF2BP2, RPSAP52, GRIP1, RN7SKP15, CCND2, CCND2-AS1, PROX1, PROX1-AS1, UBE2E2, ANK1, NKX6-3,  RBM33, GTF3AP5, PPP2R2C, WFS1, MIR4432HG, GPSM1, HNF1B, RPL26P19, C5orf67, MTNR1B, LINGO1, HMG20A, CDC123, RN7SL198P, CTBP1, THADA, ADCY5, MIR4686, ASCL2, TCF7L2, KCNQ1, CDKAL1, DMRTA1, CDKN2B-AS1, ABCC8, MACF1, SLC30A8,  HHEX, ZNF800, JAZF1, NYAP2, MIR5702, GIPR, RN7SL836P, CTRB2, CTRB1, SPRY2, LINC01080, ARPIN-AP3S2, AP3S2, RNF6, PPIP5K2, MACIR, LINC01991, LPP-AS2, RPS3AP49, RNU4-17P, ARAP1, RPL35AP3, UBE3C, TOMM22P3, KL, POU5F1,  TCF19, PDXDC1, PDE3A, SMARCC1, HPSE2, MDM4, LINC00681, LONRF1, GMEB1,  YTHDF2, POP7, EPO, SORBS2, ATP8B2, RPSAP17, USP44, SV2A, SF3B4, TSC22D2,  LINC01214, HHIP, LINC00838, EBF2, LRRC1, MLIP, ACVR1C, CDKN1B, SUMO2P3,  CICP11, LIN7A, ANO6, ARNTL, EML6, OR7K1P, LINC02306, ZBED3-AS1, ATXN7, RPS3AP18, TMEM154, RREB1, PRC1-AS1, PRC1, GLIS3, ANKH, PRDX4P1, THAP12P9, ARL15, ZZEF1, NPM1P47, C2CD4B, GCK, FAF1, HNF4A, LINC02245, LINC02576, MIR17HG, PPIAP23, NRXN3, ABO, AGPAT1, PPT2-EGFL8, BCL2, HNF1A, IGF2BP1, GIP, SLC2A2, APOE, PTGFRN, CCND1, AUTS2, ANKDD1B, FGFR3, TACC3, ST6GAL1, TFAP2B, MIR588, HSD17B1, HSD17B1-AS1, TMEM18, Y_RNA, TM6SF2, GCDH, SYCE2, NOTCH2, PEPD, PTPN9, DLEU1, DLEU7, RSPO3, ZC3H11B, NFAT5, RASGRP1, SLC12A8, FAM234A, LINGO2, NDUFAF6, SLC39A11, NSD1, MIR3168, RN7SL597P, RNU6-526P, ZNF10, SETD5, TM4SF4, WWTR1, UNC5C, LTK, SRGAP2, GINS2, BMPR2, RN7SL40P, LINC00910, PLEKHM2, ARVCF, ZNF239, ZNF487, PKHD1, STRC, CKMT1B, LINC01556,, KRT18P1, LRMDA, BIN3, CPNE4, L3MBTL2, EP300-AS1, EMSY, PDIK1L, TRIM63, APIP, TEX41, LPL, RPL30P9, DDC, FIGNL1, COL27A1, VWA8P1, PIK3R1, GBA2, ASTN2, TSEN15, COLGALT2, ZFPM1, LRFN2, LDHB, DNM3, PIGC, KLHDC4, SLC7A5, TPCN2  CAST, PCSK1, PLEKHM3, FOCAD, EGFEM1P, SGIP1, BDNF-AS, BDNF, GRP, SEC11C, CNTN2, CEP120, MIR4435-2HG, BCL2L11, ZNF236, TMEM106B, LINC00824, PGM1, JADE2, ZC3H4, MFHAS1, ALKBH3, HSD17B12, BNIPL, QSER1, HAUS6, RBMS1, ETV1, RBMX2P4, ZBTB46, TCF12, RPL19P16, LINC01153, LINC02451, ERN1, ATP2A1, IFT52, PCGF3, LINC01524, CLEC14A, MYO5C, DGKD, CCDC92, H4P1, KLF14, ACSL1, SINHCAF, CEBPB, PELATON, LINC02537, PIM3, MSRA, TRIM66, NEUROG3, TMEM256P1, CDK2AP1, C12orf65, SLC22A3, RALY, TENT5C, MBNL1, MOB1B, TMEM219, FRAT1, FRAT2, ZBTB38, XKR6, LINC00529, ETS1, LINC01122, PNPLA3, ADAMTS9-AS2, KSR2, PLEKHA1, WSCD2, CHCHD2P9, LTBP3, MAP2K7, LRRC8E, HSF1, CCDC9B, PHGR1, ABCB10, TCF4, CRHR2, CMIP, ITPR2, SSPN, BPTF, FBRSL1, EBF1, KLHL21, EYA2, TRPS1, TSPAN8, NEPNP, NUS1, LINC01625, ATP5PBP6, KCNU1, SMARCE1P4, RPS6P12,  GALNT3, JMJD1C, ZBTB26, H3P29, LINC01230, IPO9, PURG, TEX15, ZNF799, BEND3, RNU6-1299P, RPL37P6, LINC00968, MAP2K5, NMI, TNFAIP6, MAML3, MED23, PKLR, MRAS, NME9, BRAF, PARP8, WBP1L, RNU6-1231P, DNAJC2, PSMC2, CWH43, ZNRF3, NLGN1, GRID1, OR5B1P, OR5B17, ABCC5, EEF1A1P8, SPHKAP, ACTN1-AS1, RPS29P1, TSHZ3-AS1, TRAF3, CDKL2, ODAPH, RPL13, ERLIN1, LINC00558, ZNF646P1, SEPHS1P2, LINC02838, TWF1P1, CRYBA1, CBX1, UHRF1, KDM4B, LINC02224, BTD, BRD3OS, SBF2, LAMA1, JARID2, DNAH7, STK17B, FADS2, OR5L2, OR5D18, INKA2, INKA2-AS1, ADRB1, RNU6-709P, MYO19, LARP1B, HOXC4, ZNF76, MARVELD3, TAT-AS1, RPTOR, LHFPL3, MIR30B, ZFAT, EYA1, CASC11, RN7SL786P, DGAT2, IRX3, LINC02140, STAU2, EEF1B2P5, FAM227B, KCNS3, PARD3B, PLA2G6, MAFF, ZNF746, ZNF767P, CUL1, ALDH1A2, KIF3C, NFIB, IGF1R, VPS53, IKZF2, MIR4776-1, NUDT3, RPL35P2, GSAP, GCNT1P5, SUGCT, EPB41L4B, RDH14, CRTC1, HMGB3P15, ZFHX3, CASR, HEATR5B, BEND7, LINC00907, PTPRD, MNAT1, COX5BP8, SOAT1, LEPR, MFSD4B, REV3L, TMEM87B, ZHX3, HTT, FSD2, FCGRT, LINC01605, RAB3C, LBX1-AS1, RNU2-43P, KDM3A, CNTNAP2, SP9, LINC01305, SLC39A8, MAGI2, SAMMSON, CTTNBP2, PSMA3-AS1, ARMH4, PSMA3, HSPA12A, HLA-G, HCP5B, AFF3, LINC01102, TTN, CCDC88B, PRDX5, PNKD, KCTD8, LINC02465, MGAT1, PI16, C6orf89, MAML2, HS6ST3, UBE2O, PATJ, MIR375, CRYBA2, RFT1, XYD6-FXYD2, FXYD2, ZC3H13, SIAH3, KPNA3, RNY4P30, SIDT1, FOXK1, TMEM161B-AS1, RBMS3, NOS1, ERBB4, TOM1, SYNDIG1L, NPC2, CGGBP1, ZNF654, MDGA2, CHD4, ADAMTSL3, GLP2R, CACNA2D3, LINC02523, HEY2, LINC00862, CCNQP1, TSHZ2, HMGB1, UBE2L5, GUCY1B1, ARPP21, SUPT3H, CAPZA1P4, PRKD1, ELP3, RPL5P22, NOL4, MTND2P8, SH3D21, EVA1B, LINC02144, CHD1, LMBRD2, UGT3A2, RFX3, KIAA1522, SYNC, H4C5, H2BC8, CALCR, AZIN1, EP400, GDF6, GAPDHP30, SLC41A1, PM20D1, FOXP1, EIF4E3, SLC25A12, HAT1, LINC02622, PCBD1, MPPED2-AS1, L3MBTL3, TMEM244, ARID5B, NELL1, AOAH, ROBO2, FAM13A, SGCZ, SEC23IP, GACAT3, LINC02641, CCM2, NACAD, AOPEP, GTF2I, KCNJ12, WDR7, LINC02790, MED27, LAMC1, KDF1, TRPV5, SLC1A2, FIBCD1, HSP90B3P, WDR82P2, CAMK2G, SHBG, PRDM5, LINC02337, UNC79, LINC02338, SLIT2, DEPDC5, ART3, SHROOM3, NME5, WNT8A, RANBP17, SRP54-AS1, MARK3, MED20, USP49, KCNH7, STK35, ENO3, HMBS, VPS11, SLC38A9, TCF3, AMFR, NUDT21,LNC-LBCS, LINC01933, GLRA1, IL34 |
| GCST010553  (107) | HLA-DQB1, GLP1R, NEPNP, RPL35AP3, ETV1, DGKB, RNA5SP230, AUTS2, PAX4, CPA1, UBE3C, NKX6-3, SLC30A8, GLIS3, CDKN2B-AS1, TLE1, GPSM1, CDC123, TMEM256P1, LRMDA, ZMIZ1, HHEX, CPN1, TCF7L2, LINC01153, IGF1R, USP48, FAF1, TMEM18, RAMP2, MC4R, BCL2, ZNF257, GIPR, HNF4A, NFATC2, PLEKHA1, KCNQ1, SLC1A2, ARAP1, ETS1, RN7SKP15, SINHCAF, WSCD2, KSR2, SPPL3, RNF6, TOMM22P3, DLEU1, KL, RASGRP1, CCDC9B, C2CD4B, HMG20A, ARPIN-AP3S2, PRC1-AS1, GP2, FTO, ZZEF1, SLC16A11, HNF1B, GCKR, LINC01122, SCTR, EPC2, GRB14, DGKD, PPARG, UBE2E2, PSMD6, CASR, IGF2BP2, CTBP1, TACC3, PPP2R2C, PRDX4P1, MOB1B, RNU6-891P, TMEM154, PARP8, ITGA1, C5orf67, ZNF346, RREB1, CDKAL1, HLA-C, TSPAN15, RN7SL198P, RNU6-1035P, MYL7, MTCO3P1, NUS1, Y_RNA, RBMX2P4, LINC01875, RNU4-17P, RN7SL836P, PHGR1, NPM1P47, AP3S2, PRC1, PSMD6-AS1, FGFR3, THAP12P9, RPS3AP18, FGFR4, HCG27 |
| GCST007515  (36) | HNF1A, HNF1A-AS1, RREB1, MACF1, MINDY1, GCKR, THADA, CEP68, PPARG, SENP2, WFS1, ANKH, POC5, PAM, TCF19, PAX4, LPL, SLC30A8, GPSM1, KCNJ11, TPCN2, ARAP1, MRPS35, WSCD2, SBNO1, ZZEF1, MLX, TTLL6, C17orf58, TM6SF2, ZHX3, HNF4A, HORMAD2-AS1, MTMR3, PNPLA3, PIM3 |
| GCST007516  (34) | CEP68, PPARG, KIF9-AS1, KIF9, SENP2, WFS1, ANKH, PAM, RREB1, TCF19, PAX4, LPL, SLC30A8, GPSM1, KCNJ11, PLCB3, TPCN2, ARAP1, MRPS35, WSCD2, SBNO1, ZZEF1, TM6SF2, GIPR, HNF4A, ASCC2, PNPLA3, PIM3, HNF1A, HNF1A-AS1, MACF1, MINDY1, GCKR, THADA |
| GCST006867  (174) | MBNL1, CDKN2C, PTGFRN, NOTCH2, PROX1, LYPLAL1-AS1, ABCB10, NUDT6, GCKR, THADA, MIR4432HG, C5orf67, POC5, ZBED3-AS1, PAM, NYAP2, SPHKAP, PPARG, CDKAL1, NFKBIL1, HLA-DQB1-AS1, HLA-DOB, MACF1, TNIK, IGF2BP2, ST6GAL1, LPP-AS2, WFS1, TMEM18, SCD5, SLC9B2, LINC01875, FBXW7, ACSL1, ANKH, ARL15, LINC02245, MIR4435-2HG, Y_RNA, RBMS1, COBLL1, YTHDC2, SSR1, UBE2E2, PTH1R,  SCAANT1, ADAMTS9-AS2, ADCY5, TSC22D2, RPSAP52, HMGA2, TSPAN8, SOCS2, OASL, PITPNM2, RFLNA, TOMM22P3, DLEU7, SPRY2, NRXN3, C2CD4B, USP3, LINGO1, AP3S2, PRC1-AS1, FAM234A, FTO, NFAT5, CTRB2, CMIP, ZZEF1, RAI1, HNF1B, STAT3, OSBPL7, UBE2Z, IGBP1P2, MAP3K3, LAMA1, RNU4-17P, SUGP1, RN7SL836P, LINC00261, RALY, HNF4A-AS1, EYA2, HORMAD2, SAMM50, LINC02537, RPS17P5, MED23, ARG1, SLC22A2, DGKB, GTF3AP5, JAZF1, YKT6, RELN, CTTNBP2, KLF14, UBE3C, FAM86B3P, RNU6-526P, PINX1, RPL30P9, MIRLET7A1HG, ABO, SLC30A8, ZNF34, GLIS3, ZMIZ1, CHUK, TCF7L2, PLEKHA1, KCNQ1, KCNJ11, ETS1, ARAP1, MTNR1B, ETS1-AS1, CCND2, ITPR2, PTHLH, DMRTA1, UBAP2, RNU6-1035P, TLE1, PURG, SMARCE1P4, ANK1, NDUFAF6, CDC123, RPL5P26, CAMK2G, CDKN2B, SSPN, RN7SKP15, CDKN2B-AS1, KCNU1, RN7SL198P, LINC02651, MIR4421, PROX1-AS1, RPL26P19, MIR5702, HLA-DQB1, LINC01991, SLC2A2, LINC02576, LINC01101, ATXN7, LINC01214, CRADD, KL, DLEU1, LINC01080, NPM1P47, HMG20A, ANPEP, PRC1, CTRB1, CAVIN1, TEX14, TACO1, GIPR, HNF4A, VEGFA, TFAP2B, SLC22A3, H4P1, PRAG1, RNU6-1151P, PTPDC1, LPL |
| GCST010272  (15) | NYAP2, MIR5702, COBLL1, GCKR, SYN2, ARL15, H4P1, KLF14, GLIS3, Y_RNA, EXOC6, CDC123, RN7SL198P, TCF7L2, KCNJ11 |
| GCST011337  (12) | ADCY5, C5orf67, MIR588, CENPW, H4P1, KLF14, TCF7L2, ARAP1, ZC3H11B, COBLL1,  PPARG, CDC123 |
| GCST011329  (14) | MTOR, FAF1, COBLL1, PPARG, ADAMTS9-AS2, ADCY5, IGF2BP2, ARL15, JAZF1, TCF7L2, NCR3LG1, NUCB2, ARAP1, BCL2 |
| GCST011321  (3) | PPARG, C5orf67, RPL26P19 |
