# Supplementary material for: In silico identification and functional prediction of differentially expressed genes in South Asian populations associated with type 2 diabetes
Source: PLoS One. 2023 Dec 14;18(12):e0294399. doi: 10.1371/journal.pone.0294399 (PMC10721103; doi:10.1371/journal.pone.0294399)
Supplement: S3 Table — (DOCX) [file pone.0294399.s004.DOCX]

| **S3 Table.** The top 10 most significant gene ontology (GO) terms | | | | | |
| --- | --- | --- | --- | --- | --- |
| **Category**  **(GO)** | **ID** | **Name** | **p-value** | **Genes from**  **Input** | **Genes in**  **Annotation** |
| **Biological Processes** | GO:0051254 | Positive regulation of RNA metabolic process | 5.13E-16 | 146 | 1844 |
|  | GO:0010557 | Positive regulation of macromolecule biosynthetic process | 6.55E-16 | 154 | 1996 |
|  | GO:1903508 | Positive regulation of nucleic acid-templated transcription | 7.63E-16 | 140 | 1744 |
|  | GO:0045893 | Positive regulation of transcription, DNA-templated | 7.63E-16 | 140 | 1744 |
|  | GO:1902680 | Positive regulation of RNA biosynthetic process | 8.00E-16 | 140 | 1745 |
|  | GO:0032940 | Secretion by cell | 3.62E-13 | 120 | 1517 |
|  | GO:0030072 | Peptide hormone secretion | 4.10E-13 | 44 | 313 |
|  | GO:0045595 | Regulation of cell differentiation | 5.75E-13 | 138 | 1859 |
|  | GO:0046903 | Secretion | 1.19E-12 | 127 | 1672 |
|  | GO:0140352 | Export from cell | 1.76E-12 | 121 | 1571 |
| **Cellular Component** | GO:0035580 | Specific granule lumen | 1.27E-07 | 14 | 62 |
|  | GO:0005667 | Transcription regulator complex | 2.75E-06 | 44 | 518 |
|  | GO:0034774 | Secretory granule lumen | 2.96E-06 | 32 | 324 |
|  | GO:0042383 | Sarcolemma | 3.29E-06 | 22 | 179 |
|  | GO:0060205 | Cytoplasmic vesicle lumen | 3.84E-06 | 32 | 328 |
|  | GO:0031983 | Vesicle lumen | 4.36E-06 | 32 | 330 |
|  | GO:0099503 | Secretory vesicle | 8.17E-06 | 75 | 1113 |
|  | GO:0000785 | Chromatin | 1.67E-05 | 83 | 1294 |
|  | GO:0030141 | Secretory granule | 1.67E-05 | 63 | 905 |
|  | GO:0045202 | Synapse | 1.94E-05 | 97 | 1582 |
| **Molecular Functions** | GO:0008134 | Transcription factor binding | 6.00E-08 | 63 | 740 |
|  | GO:0017046 | Peptide hormone binding | 1.55E-07 | 14 | 61 |
|  | GO:0140297 | DNA-binding transcription factor binding | 1.83E-07 | 43 | 438 |
|  | GO:0019900 | Kinase binding | 3.33E-07 | 74 | 969 |
|  | GO:0019901 | Protein kinase binding | 8.46E-07 | 67 | 868 |
|  | GO:0001216 | DNA-binding transcription activator activity | 1.66E-06 | 46 | 524 |
|  | GO:0016301 | Kinase activity | 1.74E-06 | 70 | 940 |
|  | GO:0004672 | Protein kinase activity | 3.14E-06 | 59 | 759 |
|  | GO:0001228 | DNA-binding transcription activator activity, RNA polymerase II-specific | 3.15E-06 | 45 | 520 |
|  | GO:0042803 | Protein homodimerization activity | 4.48E-06 | 56 | 715 |
