# Supplementary material for: In silico identification and functional prediction of differentially expressed genes in South Asian populations associated with type 2 diabetes
Source: PLoS One. 2023 Dec 14;18(12):e0294399. doi: 10.1371/journal.pone.0294399 (PMC10721103; doi:10.1371/journal.pone.0294399)
Supplement: S4 Table — (DOCX) [file pone.0294399.s005.DOCX]

| **S4** **Table.** Target gene - miRNA regulatory networks | | |
| --- | --- | --- |
| **Gene ID** | **Degree** | **MicroRNA** |
| CCND1 | 396 | hsa-mir-15a-5p |
| CCND2 | 365 | hsa-mir-15a-5p |
| IGF1R | 359 | hsa-mir-16-5p |
| FOXK1 | 357 | hsa-mir-15a-5p |
| NFIC | 355 | hsa-mir-15a-5p |
| KMT2D | 336 | hsa-mir-15a-5p |
| SLC7A5 | 331 | hsa-mir-15a-5p |
| SON | 321 | hsa-mir-16-5p |
| SETD5 | 270 | hsa-mir-16-5p |
| ALDOA | 267 | hsa-mir-24-3p |
| CDKN1B | 261 | hsa-mir-24-3p |
| WNK1 | 258 | hsa-mir-19a-3p |
| DDX6 | 255 | hsa-mir-16-5p |
| MBNL1 | 253 | hsa-mir-17-5p |
| NFAT5 | 251 | hsa-mir-17-5p |
| GATA6 | 247 | hsa-mir-20a-5p |
| IGF2BP1 | 241 | hsa-let-7a-5p |
| NUDT3 | 241 | hsa-mir-16-5p |
| TSC22D2 | 240 | hsa-mir-15a-5p |
| SBNO1 | 237 | hsa-mir-15a-5p |
| NSD1 | 235 | hsa-mir-27a-3p |
| TP53INP1 | 234 | hsa-mir-17-5p |
| HMGA2 | 220 | hsa-mir-15a-5p |
| NFIB | 216 | hsa-mir-17-5p |
| ZFHX3 | 214 | hsa-mir-16-5p |
| PTEN | 213 | hsa-mir-17-5p |
| RREB1 | 209 | hsa-mir-27a-3p |
| VEGFA | 207 | hsa-mir-15a-5p |
| NOTCH2 | 201 | hsa-mir-15a-5p |
| WASF2 | 198 | hsa-mir-24-3p |
