# Supplementary material for: In silico identification and functional prediction of differentially expressed genes in South Asian populations associated with type 2 diabetes
Source: PLoS One. 2023 Dec 14;18(12):e0294399. doi: 10.1371/journal.pone.0294399 (PMC10721103; doi:10.1371/journal.pone.0294399)
Supplement: S5 Table — (DOCX) [file pone.0294399.s006.DOCX]

| **S5 Table.** Drug-gene interaction networks | |
| --- | --- |
| **Gene Name** | **Targeted Drug** |
| ABCC8 | NATEGLINIDE, GLIPIZIDE, CHLORPROPAMIDE, TOLAZAMIDE, ACETOHEXAMIDE, REPAGLINIDE, TOLBUTAMIDE, TOLBUTAMIDE SODIUM, GLYBURIDE, GLIMEPIRIDE |
| ACE | TRANDOLAPRIL, PERINDOPRIL ERBUMINE, ENALAPRILAT, PERINDOPRIL ARGININE, QUINAPRIL, HYDROCHLORIDE, FOSINOPRIL, MOEXIPRIL HYDROCHLORIDE, MOEXIPRIL, CAPTOPRIL, SPIRAPRIL, PERINDOPRIL, FOSINOPRIL SODIUM, BENAZEPRIL, ENALAPRIL, RAMIPRIL, SPIRAPRIL HYDROCHLORIDE, IMIDAPRIL, CILAZAPRIL, LISINOPRIL, ENALAPRIL MALEATE, BENAZEPRIL, HYDROCHLORIDE |
| ACHE | PRALIDOXIME, RIVASTIGMINE, ECHOTHIOPHATE IODIDE, PRALIDOXIME CHLORIDE, NEOSTIGMINE, GALANTAMINE, GALANTAMINE HYDROBROMIDE, HEXAFLUORENIUM BROMIDE, TACRINE HYDROCHLORIDE, RIVASTIGMINE TARTRATE, PYRIDOSTIGMINE, ECHOTHIOPHATE, EDROPHONIUM CHLORIDE, DONEPEZIL, TACRINE, EDROPHONIUM, PYRIDOSTIGMINE BROMIDE, AMBENONIUM CHLORIDE, ISOFLUORPHATE, DEMECARIUM BROMIDE, PHYSOSTIGMINE, NEOSTIGMINE METHYLSULFATE, DONEPEZIL HYDROCHLORIDE |
| ADRB1 | PROPAFENONE, LEVOBETAXOLOL, LABETALOL HYDROCHLORIDE, NEBIVOLOL HYDROCHLORIDE, HYDROXYAMPHETAMINE HYDROBROMIDE, METOPROLOL, ARFORMOTEROL, NEBIVOLOL, LEVOBETAXOLOL HYDROCHLORIDE, ACEBUTOLOL, INDACATEROL, NOREPINEPHRINE BITARTRATE, CARVEDILOL, OXPRENOLOL HYDROCHLORIDE, DOBUTAMINE HYDROCHLORIDE, BETAXOLOL, PROPRANOLOL HYDROCHLORIDE, TIMOLOL MALEATE, PRACTOLOL, ISOPROTERENOL HYDROCHLORIDE, EPINEPHRINE, SOTALOL HYDROCHLORIDE, TIMOLOL, XAMOTEROL, ACEBUTOLOL HYDROCHLORIDE, DOPAMINE HYDROCHLORIDE, CARVEDILOL PHOSPHATE, SOTALOL, NADOLOL, PINDOLOL, BETAXOLOL HYDROCHLORIDE, ATENOLOL, PROPAFENONE HYDROCHLORIDE, ISOPROTERENOL, EPINEPHRINE BITARTRATE, MIRABEGRON, DOBUTAMINE, LEVOBUNOLOL, LEVOBUNOLOL HYDROCHLORIDE, DROXIDOPA, DIPIVEFRIN HYDROCHLORIDE, LABETALOL, CARTEOLOL HYDROCHLORIDE |
| ADRB3 | EPINEPHRINE, CARTEOLOL HYDROCHLORIDE, CARVEDILOL PHOSPHATE, NOREPINEPHRINE BITARTRATE, EPINEPHRINE BITARTRATE, LEVOBUNOLOL, CARVEDILOL, PROPRANOLOL, TERTATOLOL, ISOPROTERENOL HYDROCHLORIDE, DROXIDOPA, NADOLOL, HYDROXYAMPHETAMINE HYDROBROMIDE, LABETALOL HYDROCHLORIDE, NEBIVOLOL, MIRABEGRON, DIPIVEFRIN HYDROCHLORIDE, ISOPROTERENOL |
| AKT2 | EVEROLIMUS |
| BAX | BORTEZOMIB |
| BCL2 | VENETOCLAX, BORTEZOMIB |
| BCL2L1 | VENETOCLAX |
| BRAF | DABRAFENIB, VEMURAFENIB, DASATINIB, SORAFENIB TOSYLATE, SORAFENIB, DABRAFENIB MESYLATE, REGORAFENIB, ENCORAFENIB, TEMSIROLIMUS |
| CACNA2D3 | BEPRIDIL HYDROCHLORIDE |
| CALCR | CALCITONIN HUMAN, CALCITONIN SALMON RECOMBINANT, PRAMLINTIDE ACETATE, PRAMLINTIDE, CALCITONIN |
| CAMK2G | BOSUTINIB |
| CASR | CINACALCET HYDROCHLORIDE, VELCALCETIDE |
| CCND1 | PALBOCICLIB |
| CDK8 | PONATINIB, SORAFENIB |
| CUL4A | LENALIDOMIDE, POMALIDOMIDE, THALIDOMIDE |
| DDC | CARBIDOPA, BENSERAZIDE |
| ERBB4 | VANDETANIB, ACALABRUTINIB, OSIMERTINIB MESYLATE, GEFITINIB, IBRUTINIB, AFATINIB DIMALEATE, DACOMITINIB |
| FGFR3 | RABEPRAZOLE SODIUM, PAZOPANIB HYDROCHLORIDE, PONATINIB, NINTEDANIB ESYLATE, ERDAFITINIB, NINTEDANIB |
| FGFR4 | NINTEDANIB, NINTEDANIB ESYLATE, ERDAFITINIB |
| FXYD2 | ACETYLDIGITOXIN, DIGOXIN, DESLANOSIDE, DIGITOXIN |
| GLP1R | ALBIGLUTIDE, LIXISENATIDE, EXENATIDE, DULAGLUTIDE, GLUCAGON, LIRAGLUTIDE, SEMAGLUTIDE |
| GLP2R | TEDUGLUTIDE |
| GLRA1 | COLCHICINE, NIFEDIPINE, TROPISETRON |
| GUCY1A1 | NITROGLYCERIN, ISOSORBIDE MONONITRATE, RIOCIGUAT, NITRIC OXIDE, ISOSORBIDE DINITRATE, SODIUM NITROPRUSSIDE |
| GUCY1B1 | SODIUM NITROPRUSSIDE, NITROGLYCERIN, RIOCIGUAT, NITRIC OXIDE, ISOSORBIDE MONONITRATE, ISOSORBIDE DINITRATE |
| HDAC11 | PANOBINOSTAT LACTATE, PANOBINOSTAT, VORINOSTA, ROMIDEPSIN, BELINOSTAT |
| HDAC9 | ROMIDEPSIN, VORINOSTAT, PANOBINOSTAT, BELINOSTAT |
| HTT | AMITRIPTYLINE HYDROCHLORIDE, CLOMIPRAMINE HYDROCHLORIDE, FLUVOXAMINE MALEATE, ESCITALOPRAM OXALATE, AMOXAPINE, DESVENLAFAXINE, PAROXETINE HYDROCHLORIDE, DESVENLAFAXINE SUCCINATE, DULOXETINE HYDROCHLORIDE, PROTRIPTYLINE HYDROCHLORIDE, FLUOXETINE HYDROCHLORIDE, VORTIOXETINE HYDROBROMIDE, NORTRIPTYLINE HYDROCHLORIDE, IMIPRAMINE HYDROCHLORIDE, VILAZODONE HYDROCHLORIDE, VENLAFAXINE HYDROCHLORIDE, MAZINDOL, TRAZODONE HYDROCHLORIDE, SERTRALINE HYDROCHLORIDE, NEFAZODONE HYDROCHLORIDE, PAROXETINE MESYLATE, CITALOPRAM HYDROBROMIDE, LEVOMILNACIPRAN HYDROCHLORIDE |
| IGF1R | MECASERMIN RINFABATE, BRIGATINIB, MECASERMIN, CERITINIB |
| INSR | INSULIN PURIFIED BEEF, INSULIN SUSP PROTAMINE ZINC PURIFIED BEEF, INSULIN DETEMIR, INSULIN SUSP ISOPHANE RECOMBINANT HUMAN, INSULIN ZINC SUSP PURIFIED BEEF, INSULIN SUSP ISOPHANE SEMISYNTHETIC PURIFIED HUMAN, INSULIN ZINC SUSP PROMPT PURIFIED PORK, INSULIN, INSULIN SUSP ISOPHANE BEEF, INSULIN ZINC SUSP EXTENDED BEEF, INSULIN GLARGINE, BRIGATINIB, INSULIN PURIFIED PORK, INSULIN GLULISINE, INSULIN SUSP ISOPHANE BEEF/PORK, INSULIN SUSP PROTAMINE ZINC BEEF/PORK, INSULIN ZINC SUSP PURIFIED BEEF/PORK, INSULIN SUSP PROTAMINE ZINC PURIFIED PORK, INSULIN ASPART, INSULIN LISPRO PROTAMINE RECOMBINANT, INSULIN ZINC SUSP RECOMBINANT HUMAN, INSULIN ZINC SUSP PROMPT BEEF, INSULIN SUSP ISOPHANE PURIFIED BEEF, INSULIN ZINC SUSP EXTENDED RECOMBINANT HUMAN, INSULIN PORK, INSULIN ZINC SUSP EXTENDED PURIFIED BEEF, INSULIN ZINC SUSP BEEF, CERITINIB, INSULIN DEGLUDEC, INSULIN LISPRO, INSULIN ZINC SUSP PURIFIED PORK, INSULIN SUSP ISOPHANE PURIFIED PORK, INSULIN ASPART PROTAMINE RECOMBINANT, INSULIN ZINC SUSP SEMISYNTHETIC PURIFIED HUMAN |
| ITGA2B | ABCIXIMAB, EPTIFIBATIDE |
| JAK1 | TOFACITINIB CITRATE, RUXOLITINIB, BARICITINIB, RUXOLITINIB PHOSPHATE, TOFACITINIB |
| KCNA6 | DALFAMPRIDINE, GUANIDINE HYDROCHLORIDE |
| KCNH7 | DALFAMPRIDINE, GUANIDINE HYDROCHLORIDE |
| KCNJ11 | TOLBUTAMIDE SODIUM, STERILE, GLYBURIDE, NATEGLINIDE, REPAGLINIDE, CHLORPROPAMIDE, GLIPIZIDE, TOLAZAMIDE, ACETOHEXAMIDE, MINOXIDIL, NICORANDIL, TOLBUTAMIDE, GLIMEPIRIDE |
| KCNK16 | GLIMEPIRIDE, QUINIDINE, OXIDE, ISOFLURANE |
| KCNK17 | NITRIC OXIDE |
| KCNQ1 | DALFAMPRIDINE, GUANIDINE HYDROCHLORIDE, EZOGABINE |
| KCNS3 | DALFAMPRIDINE, GUANIDINE HYDROCHLORIDE |
| KCNU1 | QUINIDINE |
| LEPR | METRELEPTIN |
| LTK | GILTERITINIB |
| MAP2K5 | COBIMETINIB, TRAMETINIB, BINIMETINIB, TRAMETINIB, BINIMETINIB, COBIMETINIB |
| MC4R | AFAMELANOTIDE, CORTICOTROPIN, BREMELANOTIDE |
| MTNR1B | RAMELTEON, AGOMELATINE, MELATONIN, TASIMELTEON |
| MTOR | SIROLIMUS, EVEROLIMUS, ALPELISIB, COPANLISIB, TEMSIROLIMUS, METFORMIN |
| NDUFAF1 | METFORMIN HYDROCHLORIDE |
| NTRK2 | LAROTRECTINIB |
| PDE3A | THEOPHYLLINE SODIUM GLYCINATE, DIPYRIDAMOLE, OXTRIPHYLLINE, MILRINONE, ANAGRELIDE, AMINOPHYLLINE, CILOSTAZOL, DYPHYLLINE, INAMRINONE LACTATE, THEOPHYLLINE, INAMRINONE,  MILRINONE LACTATE, ENOXIMONE, PENTOXIFYLLINE, ANAGRELIDE HYDROCHLORIDE |
| PDGFC | SUNITINIB |
| PIK3R1 | COPANLISIB, ALPELISIB |
| PPARG | OLSALAZINE SODIUM, DICLOFENAC, BALSALAZIDE DISODIUM, ZAFIRLUKAST, PIOGLITAZONE HYDROCHLORIDE, BEXAROTENE, MESALAMINE, PIOGLITAZONE, INDOMETHACIN |
| PRKD1 | MIDOSTAURIN |
| PSMA3 | CARFILZOMIB, IXAZOMIB CITRATE, BORTEZOMIB |
| PSMC2 | IXAZOMIB CITRATE, CARFILZOMIB, BORTEZOMIB |
| PSMD6 | BORTEZOMIB, CARFILZOMIB, IXAZOMIB CITRATE |
| PTH1R | TERIPARATIDE, ABALOPARATIDE, TERIPARATIDE ACETATE, PARATHYROID HORMONE |
| RAMP2 | PRAMLINTIDE ACETATE |
| SCTR | SECRETIN SYNTHETIC PORCINE, SECRETIN, SECRETIN SYNTHETIC HUMAN |
| TPCN2 | VERAPAMIL |
| TRPV5 | ECONAZOLE, MICONAZOLE |
| VEGFA | RANIBIZUMAB, PEGAPTANIB SODIUM, AFLIBERCEPT, BEVACIZUMAB |
